# T cell-mediated immune surveillance conferred by latent Epstein-Barr virus genes suppresses a broad spectrum of tumor formation through NKG2D-NKG2DL interactions

**DOI:** 10.3389/fimmu.2025.1597731

**Published:** 2025-06-04

**Authors:** Yuqi Jin, Yun Guo, Yohei Kawano, Megumi Sasatani, Shun Ohki, Keita Yamane, Yusei Ota, Yumi Tamura, Yusuke Sotomaru, Yoshihiro Baba, Tomoharu Yasuda

**Affiliations:** ^1^ Department of Immunology, Graduate School of Biomedical and Health Sciences, Hiroshima University, Hiroshima, Japan; ^2^ Department of Experimental Oncology, Research Institute for Radiation Biology and Medicine, Hiroshima University, Hiroshima, Japan; ^3^ Natural Science Center for Basic Research and Development, Hiroshima University, Hiroshima, Japan; ^4^ Division of Immunology and Genome Biology, Medical Institute of Bioregulation, Kyushu University, Fukuoka, Japan

**Keywords:** Epstein-Barr virus, LMP1, LMP2A, immune surveillance, radiation-induced tumor, T-ALL, Apc^Min^, NKG2D

## Abstract

Epstein-Barr virus (EBV)-infected B cells effectively induce T cell-mediated immune surveillance that suppresses the proliferation of EBV^+^ B cells and development of lymphomas. However, it remains unclear whether EBV-specific T cells are involved in the surveillance of EBV-negative general tumors. To address this issue, we induced immune surveillance by expressing key EBV antigens, LMP1 and LMP2A, in germinal center B cells and investigated the formation of non-B cell tumors. LMP1/2A mice showed a significantly reduced incidence of radiation-induced T-cell acute lymphoblastic leukemia/lymphoma (T-ALL) even in the absence of LMP antigens in tumor cells and an extended life-span compared to control mice. LMP1/2A mice showed significantly higher numbers of activated memory T cells in both CD4^+^ and CD8^+^ αβT cell fractions compared to controls, suggesting their role in the elimination of tumor cells. Despite nearly absent MHC class I expression, tumor cells were effectively killed by CD8^+^ T cells activated upon LMP1/2A-expressing B cells. Transcriptome analysis identified upregulation of the NKG2D-NKG2DL pathway, emphasizing the capacity of LMP1/2A-induced T cells in the recognition of common tumor specific antigens. Moreover, not only T-cell tumors, but also intestinal tumors caused by Apc^Min^ mutation were significantly suppressed by the LMP1/2A-induced immune surveillance. These results suggest that LMP1/2A-expression associated with EBV infection contributes to pan-tumor surveillance, implicating a beneficial aspect of EBV infection in humans and providing important insights into cancer prevention.

## 1 Introduction

Gammaherpesviruses are known to establish lifelong latent infections in their hosts (1) and can systemically enhance immune surveillance not only through antigen-specific responses but also by mechanisms such as bystander T cell activation, cross-presentation, and the stimulation of innate immune pathways (2–4). Among them, EBV a human-specific gammaherpesvirus, is of particular research interest due to its global prevalence and its ability to infect B cells and establish lifelong latency (5).

In healthy individuals, T and NK cell-mediated immune surveillance effectively suppresses EBV-infected B cells which are restricted into a small fraction of circulating B cells with minimal or no viral gene expression (6). However, as immune surveillance weakens, EBV adopts progressively different latency states. In latency I, characteristic of Burkitt lymphoma (BL), viral gene expression is highly restricted; in latency II, seen in Hodgkin’s disease (HD), a few viral genes are expressed; and in latency III, associated with post-transplant lymphoproliferative disorder (PTLD) and AIDS-related B cell lymphoma, all latent viral genes are activated (7, 8). Despite extensive research, the mechanisms by which EBV-infected cells activate immune surveillance and inhibit malignant tumor development remain to be fully elucidated.

Among the EBV-encoded proteins, latent membrane proteins LMP1 and LMP2A expressed in latency II and III are central to B cell activation and transformation. LMP1 mimics an active CD40 receptor, primarily activating the NF-κB and JNK signaling pathways (9, 10). Notably, *in vitro* studies have identified LMP1 as the only EBV latent protein capable of transforming rodent fibroblasts (11). In transgenic mouse models of EBV pathology, expression of LMP1 in B cells induced B cell proliferation that was subsequently cleared by T cells. Depletion of T cells promoted rapid expansion of LMP1-expressing B cells and lymphomagenesis (12, 13). Conditional LMP1 expression in mouse, where LMP1 is induced in a timed manner and only in a few fractions of B cells is sufficient to model acute EBV infection as seen in human infectious mononucleosis (14, 15). LMP2A, frequently co-expressed with LMP1, complements and amplifies the effect of LMP1 in latent EBV infection mouse models (15, 16). LMP2A substitutes for the B cell receptor (BCR) signaling, rescuing germinal center (GC) B cells from apoptosis caused by the loss of BCR expression due to somatic hypermutation (7, 17, 18). Moreover, LMP2A activates PI3K and MAPK signaling pathways (19), augmenting LMP1’s oncogenic potential while simultaneously eliciting stronger immune surveillance responses (15, 16, 20). These findings highlight the opposing actions of LMP1 and LMP2A, namely their dual roles in oncogenesis and immune surveillance of tumors.

Human studies have demonstrated that EBV-transformed B lymphoblastoid cell lines (LCLs) can induce a population of T cells that recognize EBV-negative B lymphoma cells *in vitro*, despite lacking viral antigen specificity (21). This suggests that EBV-induced immune surveillance may exert broader antitumor potential beyond targeting viral antigens alone. In mouse models, LMP1 expression during B cell transformation has been shown to induce a variety of non-viral antigens, including tumor-associated antigens (TAAs) and self-antigens, while also enhancing MHC class I antigen presentation and upregulating multiple costimulatory molecules such as CD70, OX40L, and 4-1BBL (12, 22, 23). These effects collectively activate a population of T cells capable of recognizing both viral and tumor-associated antigens. In addition, LMP1 promotes the expression of stress-induced ligands such as NKG2DL and Fas, activating innate immune pathways that enable NK cells and T cells to mediate non-specific cytotoxicity (12). These findings suggest that EBV-induced immune surveillance is not limited to classical MHC-restricted antigen-specific responses but may also involve broader mechanisms such as cross-presentation, bystander activation, and enhanced innate immune signaling. This broadened scope of T cell reactivity potentially enables the recognition and elimination of EBV-negative tumors, representing a cross-protective antitumor mechanism mediated by EBV. Notably, LMP2A acts as a functional cofactor of LMP1, enhancing not only its oncogenic potential but also its immunogenicity, thereby contributing to more robust immune surveillance (14–16, 24). However, direct experimental evidence demonstrating that EBV-induced immune surveillance can eliminate EBV-negative tumors remains lacking. In this study, we investigate whether immune surveillance triggered by the EBV-encoded proteins LMP1 and LMP2A can effectively suppress the development of general tumors independently of EBV antigens.

## 2 Materials and methods

### 2.1 Mouse strains

Previously described Cγ1-Cre mice were generated by targeting 129P2-derived embryonic stem (ES) cells and backcrossed to C57BL/6 mice (25). R26-LMP1/2A(iresEYFP)^flSTOP^ mice were generated by targeting C57BL/6-derived ES cells as described detail in the following section (**Supplementary Figure 1A**). Cγ1-Cre mice were crossed with R26-LMP1/2A(iresEYFP)^flSTOP^ mice to generate Cγ1-Cre^/+^; R26-LMP1/2A(iresEYFP)^flSTOP/+^ (GCB-LMP1/2) mice in which LMP1 and LMP2A are expressed with EYFP reporter in GC B cells. Cγ1-Cre^/+^ mice were used as the control to compare with GCB-LMP1/2 mice. Apc^Min/+^ mice (26) were crossed with GCB-LMP1/2A mice to generate Apc^Min/+^; Cγ1-Cre^/+^; R26-LMP1/2A(iresEYFP)^flSTOP/+^ (Apc^Min/+^LMP^KI^) mice which carry the Apc^Min/+^ mutation in GCB-LMP1/2A mice. For *in vitro* cell culture and *in vitro* killing assays, mice aged 10–20 weeks were used. In radiation treatment and Apc^Min/+^ induced intestinal tumor formation experiments, the timing of euthanasia was determined based on tumor progression and is specified in each corresponding experiment. All animals were housed under specific pathogen-free conditions and handled according to protocols approved by the Ethics Review Committee for Animal Experimentation of Hiroshima University and the institutional committee at Kyushu University.

### 2.2 Generation of R26-LMP1/2A(iresEYFP)^flSTOP^ mice

For the targeting vector construction, the 5’ homology arm (1.1 kb) and the 3’ homology arm (1.0 kb) of Gt(ROSA)26Sor were amplified by PCR from C57BL/6 mouse genome. LMP1 and LMP2A sequences from EBV B95–8 strain were tandemly arranged by flanking T2A self-cleaving peptide sequence, followed by EYFP gene with internal ribosome entry site (IRES) and a bovine growth hormone polyadenylation (BGH pA) sequence. The splice acceptor and the loxP flanked neomycin resistant stop cassette was amplified from Rosa26 targeting vector (27) and positioned upstream of LMP1 and LMP2A. PCR fragments were assembled in pBlueScript II plasmid vector using NEBuilder HiFi DNA assembly master mix (NEB) to generate R26-LMP1/2A(iresEYFP)^flSTOP^ targeting vector. PCR amplifications were performed using high fidelity KOD DNA polymerase (TOYOBO). The C57BL/6N ES cells, EGR-101, was kindly provided from Dr. Masahito Ikawa (28). 1x10^6^ C57BL/6N ES cells were electroporated with 100 ng/μl (0.61 μM) recombinant Cas9 Nuclease 3NLS (IDT #1074181), 36 ng/μl (1 μM) gR26–1 crRNA (5’- ACTCCAGTCTTTCTAGAAGA//TGG -3’, IDT): tracrRNA duplex, and 100 ng/μl pR26-LMP1/2A(iresEYFP)^flSTOP^ plasmid DNA in 100 μl of 1xOpti-MEM medium (ThermoFisher) using NEPA 21 super electroporator (NEPA GENE) and 2 mm gap cuvette. The following parameters were used for electroporation; Poring pulse (voltage 125 V; pulse length 2.5 msec; pulse interval 50 msec; number of pulses 2; decay rate 10%; polarity +) and Transfer pulse (voltage 20 V; pulse length 50 msec; pulse interval 50 msec; number of pulses 5; decay rate 40%; polarity ±). The electrical resistivity was typically 0.03 to 0.05 kΩ. Electroporated cells were selected for 6–7 days under ES medium (StemSure D-MEM, 15% fetal calf serum (FCS), L-glutamine, non-essential amino acids, β-mercaptoethanol, and LIF) containing 200 μg/ml G418 (Nacalai tesque). ES cell colonies were subjected to PCR screening to select correct clones. ES cells from selected clones were injected into Balb/c blastocysts. A chimeric male (80-90% chimerism) was crossed to C57BL/6 females to obtain germline transmitted offspring.

### 2.3 Radiation treatment

Mice (4-week-old) were subjected to total body irradiation (TBI) using a Gamma Cell 40 Exactor Research Irradiator (Best Theratronics), equipped with a 148 TBq ^137^Cs source. The irradiation was delivered at a dose rate of 770 mGy/min. Each mouse received a total of four weekly fractions of 1.6 Gy, resulting in a cumulative dose of 6.4 Gy. The absorbed dose was calibrated using a GD-302M glass dosimeter (AGC Techno Glass).

### 2.4 Monitoring and euthanasia criteria

After TBI, mice were monitored every three days for general health status, including activity level, posture, grooming, fur condition, and signs of apparent weight loss. For survival analysis experiments, mice exhibiting signs of distress—such as severe lethargy, hunched posture, ruffled fur, reduced mobility, or visually estimated weight loss—were euthanized according to humane endpoint criteria approved by the institutional animal ethics committee. Organs were collected from these moribund mice and used as end-stage disease samples for further analysis. All end-stage mice were euthanized between 15 and 30 weeks after radiation. For experiments assessing the incidence of T-ALL, all mice were euthanized at a defined time point—14 weeks after the final radiation—prior to the onset of overt clinical symptoms. This endpoint was selected to ensure consistency in evaluation and adherence to ethical standards.

### 2.5 B cell and T cell purification

Splenic B cells, CD4^+^ T cells, and CD8^+^ T cells were isolated by either magnetic bead-based negative selection or FACSAria cell sorter. For B cell purification, splenocytes were incubated with biotinylated anti-CD43 (R2/60) antibodies. For CD4^+^ T cell and CD8^+^ T cell purification, splenocytes were incubated with biotinylated antibody cocktail of anti-CD11b (M1/70), CD11c (HL3), CD49b (DX5), I-Ab (KH74), Ter119 (Ter119), Gr-1 (RB6-8C5), and TCRγδ (GL3) antibodies, combined with either anti-CD4 (GK1.5) antibodies for CD8^+^ T cell enrichment or anti-CD8 (53-6.7) antibodies for CD4^+^ T cell enrichment. Following 15 minutes incubation at 4°C, cells bound to biotinylated antibodies were captured using BD IMag™ Streptavidin Particles Plus (BD Biosciences) for 10 minutes, and the unbound negative fraction was collected. B cells with at least 95% purity and CD4^+^ and CD8^+^ T cells with at least 90% purity were subsequently used for cell culture experiments.

### 2.6 Preparation of B cells and T-ALL cell line

To establish LMP1 and LMP2A-expressing B cells (B^LMP1/2A^ cells), purified B cells from the spleens of GCB-LMP1/2A mice were cultured in 10% FBS-1640 medium and maintained at 37°C in a 5% CO_2_ humidified atmosphere for 12 days. Actively proliferating cells were harvested and cryopreserved at a concentration of 2 × 10^6^ cells/ml using a Bambanker (Nippon genetics) at -80°C. For experimental use, cells were thawed and cultured in 10% FBS-1640 medium for three days. For CD40 activated B cells (CD40 act-B cells), purified B cells from wild-type C57BL/6 mice were stimulated with 2 μg/ml anti-CD40 antibody (HM40-3, BioLegend) and 20 ng/ml recombinant mouse IL-4 (Biolegend) in 10% FBS-RPMI-1640 medium for 5 days. To establish a T-ALL cell lines, thymuses were harvested from terminal-stage T-ALL mice (over 20 weeks after the final radiation treatment). The thymic tissues were processed into a single-cell suspension and cultured in 10% FBS-1640 medium. The cells were passaged every three days. While non-malignant cells died within five passages, T cells stably propagating over 10 passages were considered as T-ALL cell line.

### 2.7 Western blotting

Cells were lysed in 1% NP-40 buffer (150 mM NaCl, 0.5 M NaF, 10 mM Tris pH 7.4, 0.5 mM EDTA, and 2 mM PMSF) containing 2mM phenylmethylsulfonyl fluoride (Nacalai Tesque) supplemented with protease inhibitors (Nacalai Tesque). Protein concentrations were measured using the Pierce™ BCA assay (Thermo Fisher Scientific). Equal amounts of protein were separated on 10% SDS–polyacrylamide gels and transferred to PVDF membranes (Bio-Rad). Membranes were blocked with 6% skim milk in TBS-TweenTM-20 (Thermo Fisher Scientific) and incubated with primary antibodies overnight at 4 °C, followed by HRP-conjugated secondary antibodies. Proteins were visualized using ECL reagents (Cytiva) and detected with ImageQuant LAS 500 (GE Healthcare). The following antibodies were used: mouse anti-EBV LMP1 (S12,Sigma-Aldrich), rat anti-EBV LMP2A (14B7, Santa Cruz Biotechnology), rabbit anti-β-actin (Proteintech), HRP-conjugated goat anti-mouse IgG (Sigma-Aldrich), HRP-conjugated donkey anti-rat IgG (Jackson ImmunoResearch), and HRP-conjugated goat anti-rabbit IgG (Proteintech).

### 2.8 *In vitro* cell culture

All cells were cultured in RPMI-1640 (FUJIFILM Wako Chemicals) medium supplemented with 10% fetal bovine serum (FBS), 1 mM sodium pyruvate, 52 μM β-mercaptoethanol, non-essential amino acids, and penicillin-streptomycin (referred to as 10% FBS-1640). For *in vitro* T cell culture, FACS-purified CD4^+^ or CD8^+^ T cells from the spleen were co-cultured with γ-irradiated (6–8 Gy) T-ALL, B^LMP1/2A^ and CD40 act-B cells at a 1:1 T cell to lymphoma cell ratio. The cultures were re-stimulated every three days in 10% FBS-1640 medium supplemented with 10 ng/mL IL-2 (R&D Systems).

### 2.9 *In vitro* killing assay

The *in vitro* killing assay was adapted from previously described methods with modifications (12, 24). Briefly, *ex vivo* expanded CD8^+^ T cells were purified by FACS sorting and incubated with target cells at varying effector-to-target (E: T) ratios for 4 hours in U-bottom 96-well plates. Prior to incubation, the effector-target cell mixtures were centrifuged at 200 rpm for 2 minutes to facilitate cell contact. Following incubation, cultures were stained with antibodies against TCRβ, CD8a, and CD24 to distinguish target and effector cells. Cell viability was assessed by propidium iodide (PI, Sigma-Aldrich) staining, where dead cells were identified as PI-positive and viable cells remained PI-negative. Percent-specific killing was calculated using the formula: % specific killing = (% apoptotic target cells in cultures with both effectors and targets) − (% apoptotic target cells in cultures with targets alone). In blocking receptor experiments, *ex vivo* expanded CD8^+^ T cells were pre-incubated with NKG2D antibody, 2B4/SLAMF4 antibody, or corresponding isotype controls at a concentration of 20 µg/ml for 60 minutes at 37°C. The killing assay was then performed at an E:T ratio of 20:1 in the continued presence of the blocking antibodies.

### 2.10 Isolation of lymphocytes from intestinal tumor nodule

To isolate lymphocytes from tumor nodules, mice were euthanized, and the intestines were excised, thoroughly rinsed, and soaked in cold PBS. Tumor nodules were collected from both the small intestine and colon, then minced and digested in a solution containing 1 mg/mL Collagenase D (Roche) and 0.5 mg/mL DNase I (Roche) at 37°C with shaking for 1 hour. The resulting cell suspension was filtered through a 70-μm nylon mesh and treated with Gey’s solution to lyse red blood cells. Cells were then washed with 2% FBS/PBS and resuspended in 33.75% Percoll (GE Healthcare Life Sciences) before centrifugation at 2,300 rpm for 25 minutes at room temperature.

### 2.11 Flow cytometry

Single-cell suspensions from the spleen, Peyer’s patches (PP), mesenteric lymph nodes (mLN), thymus, and bone marrow were resuspended in Gey’s solutions for red blood cell lysis. Cells were treated with FcBlock (2.4G2, BD Biosciences) followed by staining with fluorochrome-conjugated antibodies or biotinylated antibodies. The cells stained with biotinylated antibodies were detected by fluorochrome-conjugated streptavidin. Anti-B220/CD45R (RA3-6B2), CD19 (6D5), Notch1 (HMN1-12), CD107a (1D4B), NK-1.1 (PK136), Granzyme B (GB11), Perforin (B-D48), PD-1 CD279 (29F.1A12) were purchased from BioLegend; Anti- IFN-γ (XMG1.2), Ly-6A/E (Sca1, D7), CD117 (2B8), CD95 (Jo2), CD4 (RM4-5), CD8a (53-6.7), CD24 (M1/69), TCRβ (H57-597), CD44 (IM7), CD25 (7D4), CD69 (H1.2F3), IgG2a (R35-95) isotype control, TCR γδ (GL3) were purchased from BD Biosciences; Anti- c-Myc (AG1263) were purchased from Proteintech, Anti- CD45 (30-F11), CD314 (CX5), CD244.2 (eBio244F4) were purchased from Thermo Fisher Scientific. Cells were stained with PI (Sigma-Aldrich) to exclude dead cells. For intracellular staining, the cells were fixed and permeabilized using a Foxp3 staining kit (Thermo Fisher Scientific). For cytokine staining, cells were treated with 50 ng/ml of phorbol 12-myristate 13-acetate (PMA) and 1 μg/ml of ionomycin with BD GolgiStop containing monensin for 6 hours followed by staining using cytofix/cytoperm plus fixation/permeabilization solution kit (BD Biosciences). Stained cells were analyzed using FACSCanto II (BD Biosciences) and CytoFlex (BECKMAN COULTER) data were analyzed on FlowJo software (BD Biosciences).

### 2.12 Histology and immunohistochemistry

Cryosections (6 µm) of spleen, thymus, and intestinal tumor tissues were prepared, air-dried, and either immediately used for hematoxylin and eosin (H&E) staining or dehydrated in acetone, air-dried, and stored at -80°C for subsequent immunohistochemistry. For immunohistochemistry, the following antibodies were used: anti-Keratin 5 (AF138) from Covance Research and anti-Keratin 8 (Ks8.7) from PROGEN; anti-CD8 (53-6.7) and CD90.2 (30-H12) from BioLegend; and anti-CD4 (RM4-5) and CD45R (RA3-6B2) from BD Biosciences.

### 2.13 Microarray

Gene expression profiling was performed on FACS-sorted T cell samples using Affymetrix GeneChip Mouse Genome 430 2.0 Arrays, according to the manufacturer’s recommendations (Affymetrix, Santa Clara, CA, USA) and data analysis was performed as previously reported (24, 29, 30). Fluorescence ratios were normalized by applying the RMA algorithm using the BRB Array Tools software package (available at https://brb.nci.nih.gov/BRBArrayTools/). These data were then interpreted in comparison to gene expression results obtained in naive T cells. Heatmap were generated by using R-tidyheatmaps. The complete microarray data are available at the Gene Expression Omnibus (http://www.ncbi.nlm.nih.gov/projects/geo; accession number GSE206802).

### 2.14 Quantitative real time PCR

Total RNA was extracted from normal thymus and spleen, irradiated thymus and spleen, T-ALL cell line, B^LMP1/2A^ cells, and CD8^+^ T cells activated by T-ALL and B^LMP1/2A^ cells using ISOGEN II (NIPPON GENE) following the manufacturer’s instructions. Complementary DNA (cDNA) was synthesized using the ReverTra Ace qPCR Master Mix (TOYOBO) according to the manufacturer’s protocol. Real-time PCR was performed with Thunderbird SYBR qPCR mix (TOYOBO) on a Bio-Rad Real-Time PCR system. The specific primers used were as follows: (Notch1: 5’-GCTGCCTCTTTGATGGCTTCGA-3’ and 5’-CACATTCGGCACTGTTACAGCC-3’; Myc: 5’-TCGCTGCTGTCCTCCGAGTCC-3’ and 5’-GGTTTGCCTCTTCTCCACAGAC-3’; Lmo-2: 5’-TGGGACGGAAATTGTGCAGGAG-3’ and 5’-GGCGCATTTGAAACACTCCAGG-3’; Ptcra: 5’-CTGCAACTGGGTCATGCTTC-3’ and 5’-GTCCAAATTCTGTGGGTGGG-3’; Klrk1: 5’- CCTATCACTGGATGGGACTGGT-3’ and 5’- GCTTGAGCCATAGACAGCACAG-3’; Ulbp1: 5’- GTGCAGGAGACTAACACAACCG-3’ and 5’- TGCCAGTGCTTGTGTCAACACG-3’; Cd244: 5’- CAGTTGCCACAGCAGACTTTCC-3’ and 5’- CTTCCTGGAAGGCTGGACTACT-3’; Cd48: 5’- GCTGCGTGAAACTGAGAACGAG-3’ and 5’- CACACGATAGCCTCAGGTGACA-3’).

### 2.15 Detection of V–J recombination at the TCRβ locus

Thymocytes harvested 14 weeks after the final radiation treatment were suspended in lysis buffer containing Proteinase K (Roche) and incubated at 55°C for 1 hour, followed by 95°C for 5 minutes to inactivate the enzyme. The lysates were used as templates for PCR amplification. Primers were used as previously reported (31): Dβ1, 5’-TTATCTGGTGGTTTCTTCCAGC-3’; Dβ2, 5’-GCACCTGTGGGGAAGAAACT-3’; Jβ1.6, 5’-GGTAGAAAGGTAGAGGGTTCCAGA-3’; Jβ2.6, 5’-TGAGAGCTGTCTCCTACTATCGATT-3’.

### 2.16 Statistical analysis

All data analyses were performed with Prism 9 software (GraphPad). The results were presented as mean ± SD. p values were calculated by multiple student`s t-test, multiple t-tests with Holm-Šidák correction, one-way or two-way ANOVA. Statistical Significance was determined with alpha<0.05 and presented as *p < 0.05; **p < 0.01; ***p < 0.001; ****p < 0.0001; ns, not significant.

## 3 Results

### 3.1 Establishment of immune surveillance in GCB-LMP1/2A mice

**Figure 1:**
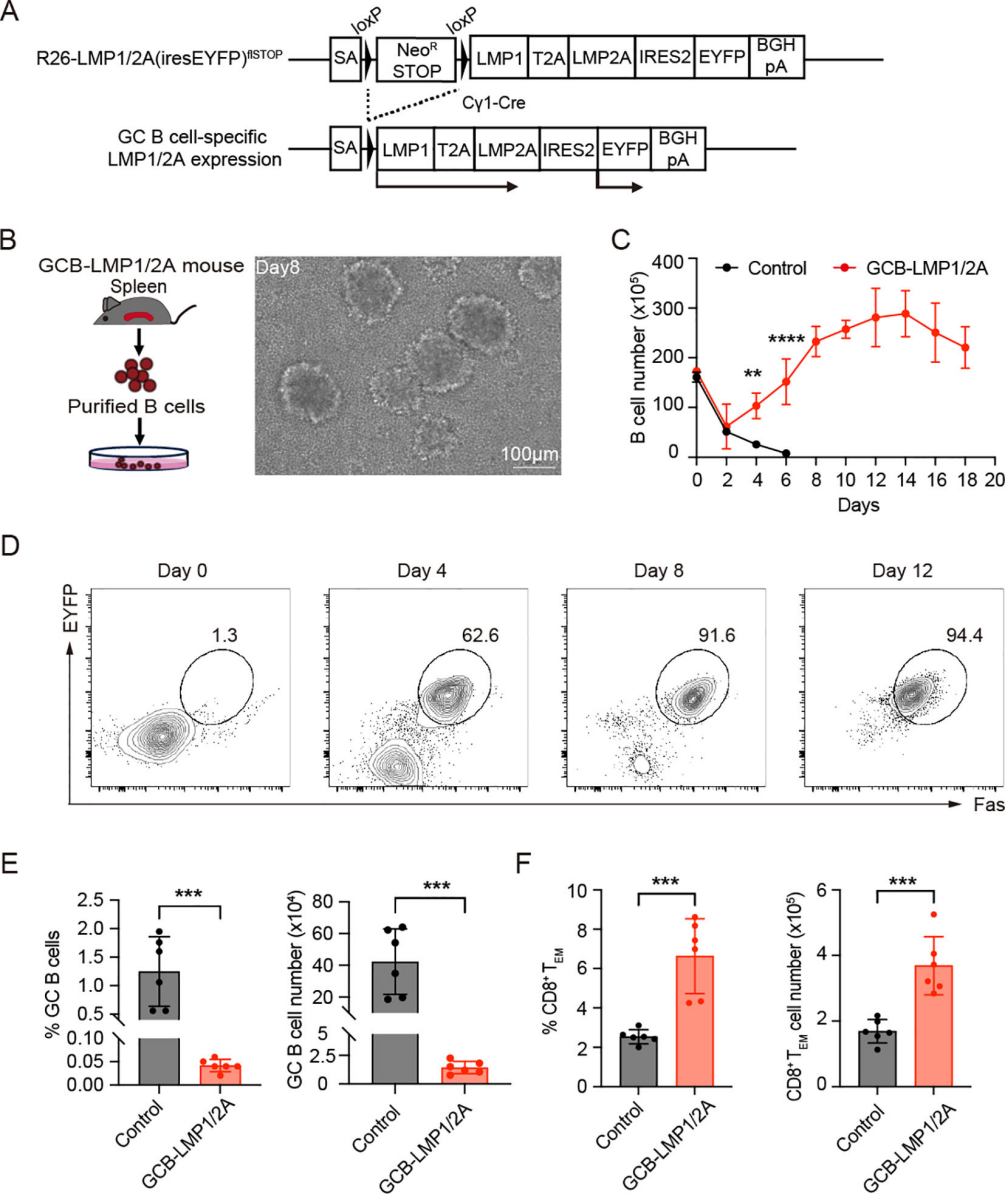
Characterization of germinal center B cell-specific LMP1/2A-expressing mice. **(A)** Targeting strategy for conditional expression of LMP1 and LMP2A in germinal center (GC) B cells. LMP1, LMP2A, and EYFP reporter genes were targeted into the Rosa26 locus with the neomycin resistance stop cassette enabling constitutive expression of those genes after Cre-mediated excision of stop cassette (R26-LMP1/2A(iresEYFP)^flSTOP^ mouse) and crossed with GC B cell-specific Cre strain, Cγ1-Cre mice. SA, splice acceptor. **(B)** Workflow for isolating and culturing LMP1/2A^+^ B cells from GCB-LMP1/2A mice (*left*). Morphological changes of B cells during culture, showing increased cell size and aggregation into large clumps (*right*). Scale bar, 100 μm. **(C)** Growth curves of *in vitro* cultured B cells from control and GCB-LMP1/2A mice (n=3). Statistical significance tested using two-way ANOVA with Bonferroni’s multiple comparisons test; **p < 0.01; ****p < 0.0001. **(D)** FACS analysis of *in vitro* cultured B cells. Proliferating cells expressed Fas, confirming their GC B cell identity. EYFP was used as a reporter for LMP1/2A expression. **(E, F)** Percentage and cell count of GC B cells **(E)** and CD8^+^ T_EM_ cells **(F)** in the spleens of GCB-LMP1/2A mice compared to control mice (n=6). Statistical significance tested using an unpaired two-tailed Student’s t-test; ***p < 0.001.

To mimic the immune surveillance induced by latent EBV infection in humans as previously reported (15, 16), we generated a genetic mouse model Cγ1-Cre^/+^; R26-LMP1/2A(iresEYFP)^flSTOP/+^ referred to hereafter as GCB-LMP1/2A mice in which LMP1 and LMP2A are expressed with EYFP reporter in GC B cells (**Figure 1A**, **Supplementary Figure 1A**). To confirm the expression and transforming potential of LMP1/2A, we purified B cells from the spleens of GCB-LMP1/2A mice and cultured them *in vitro* (**Figure 1B**). In culture, B cells expressing LMP1 and LMP2A displayed characteristic cluster formation and significant proliferative activity (**Figures 1B, C**). FACS analysis revealed that these proliferating cells co-expressed Fas, a marker for LMP1 expression (12, 32), and EYFP reporter protein, confirming that expression of LMP1 and LMP2A induces marked proliferation in B cells (**Figure 1D**). Western blot analysis further confirmed expression of LMP1 and LMP2A in B cells (**Supplementary Figure 1B**). Hereafter, we refer to *ex vivo* expanded LMP1/2A-expressing B cells as B^LMP1/2A^ cells. These findings demonstrated that LMP1 and LMP2A are successfully expressed in GC B cells and have potential transforming capacity of B cells. However, no abnormal B cell proliferation or lymphomas were observed in immune-competent GCB-LMP1/2A mice. Instead, the number and percentage of GC B cells in the spleens of GCB-LMP1/2A mice were significantly reduced compared to control mice (**Figure 1E**), while the proportion of CD8^+^ effector memory T cells (T_EM_) defined by CD8^+^CD44^+^CD62L^-^ surface phenotype was notably increased (**Figure 1F**). Similar trends were observed in the mesenteric lymph nodes (mLNs) and Peyer’s Patches (PPs), where GC reactions are consistently observed (**Supplementary Figure 1C**). To further confirm that the increase in CD8^+^ T_EM_ cells was directly induced by LMP1/2A-expressing B cells, we conducted an *in vitro* co-culture assay using CD8^+^ T cells isolated from control mice. When co-cultured with B^LMP1/2A^ cells or CD40-activated B cells (CD40 act-B), B^LMP1/2A^ cells promoted the proliferation of CD8^+^ T_EM_ cells, whereas CD40 act-B cells did not (**Supplementary Figures 1D, E**). These results indicate that immune surveillance against LMP1/2A-expressing B cells successfully established in GCB-LMP1/2A mice, effectively suppressing expansion of those B cells and maintaining CD8^+^ T cells in a memory state.

### 3.2 Total body irradiation induces T cell acute lymphoblastic leukemia/lymphoma

**Figure 2:**
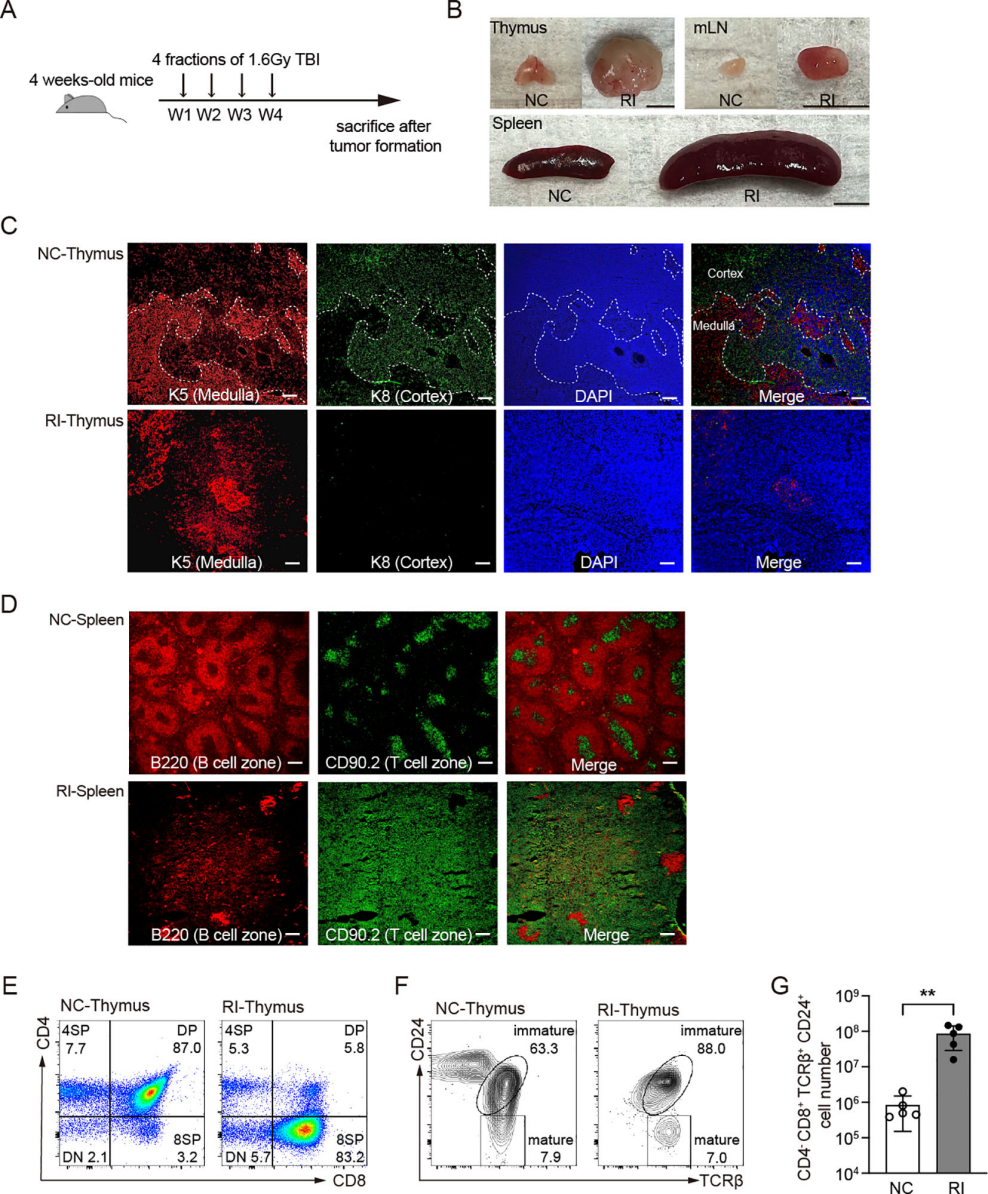
Histological and flow cytometric analysis of mice subjected to total body irradiation. **(A)** Mice received 1.6 Gy of total body irradiation (TBI) weekly at indicated time points (vertical arrows) for a total four doses of 6.4 Gy. W, week. **(B)** Representative pictures of thymus and spleen from non-irradiated control mice (NC) and mice after 30 weeks of radiation (RI). mLN, mesenteric lymph nodes. Scale bar=0.5 cm. **(C, D)** Immunostaining of thymus **(C)** and spleen **(D)** from the indicated mice. **(C)** Thymus were stained with K5 and K8 antibodies to identify thymic epithelial cells. Thymic medulla, K5^+^; Thymic cortex, K8^+^. **(D)** Splenic B cell zone and T cell zone were stained with B220 and CD90.2, respectively. Scale bar=200 μm. **(E-F)** Representative FACS results of thymus from NC and RI mice with T-ALL. **(E)** CD4 and CD8 markers distinguish stages of T cell development. **(F)** CD24 and TCRβ markers distinguish mature and immature stage from 8SP cells. **(G)** Cell number of T-ALL (CD4^-^CD8^+^TCRβ^+^CD24^+^) in thymus from NC and RI mice. Each dot indicates one mouse (n=5). Statistical significance tested using an unpaired two-tailed Student’s t-test; **p < 0.01. All RI samples were collected from mice at the end stage of disease progression, between 15 and 30 weeks after radiation, based on humane endpoint criteria detailed in the Materials and methods section.

EBV-specific cytotoxic T lymphocytes (CTLs) not only exhibit therapeutic effects against EBV-associated lymphomas but have also been found to have potential efficacy against EBV-negative tumors, particularly B cell malignancies (21, 33–36). However, it remains unclear whether it has a similar effect on EBV-negative T-cell-associated tumors. Repeated low-dose gamma-ray radiation exposure has been shown to induces thymic-origin T-cell acute lymphoblastic leukemia/lymphoma (T-ALL) in over 90% of C57BL/6 mice (37–39). In this study, we subjected 4-week-old mice to total body irradiation (TBI), administering 1.6 Gy by weekly four times, 6.4 Gy in total (**Figure 2A**). Approximately 15 weeks post-radiation, symptoms such as rickets, weight loss, and reduced mobility began to appear, which eventually progressed to T-ALL. Upon dissection, we observed abnormal enlargement of the thymus, mLN, and spleen (**Figure 2B**), accompanied by an increase in cellularity (**Supplementary Figure 2A**). Immunofluorescence staining demonstrated the disruption of normal cortical and medullary regions in the thymus, suggesting impaired thymic function and abnormal T-cell development (**Figure 2C**). The spleen showed T cell hyperplasia, accompanied by a significant reduction in B cell areas (**Figure 2D**). FACS analysis revealed that these abnormally proliferating and tissue infiltrating T cells were CD24^+^ immature T cells (**Supplementary Figure 2B**). Furthermore, no B cell hyper-proliferation was observed in GCB-LMP1/2A mice, suggesting that immune surveillance against LMP1/2A^+^ B cells remains active even after the onset of T-ALL (**Supplementary Figure 2C**).

Compared to the non-irradiated control group, irradiated mice exhibited an abnormally increased percentage of CD8 single-positive (8SP) thymic cells (**Figure 2E**). These cells displayed an immature phenotype characterized by TCRβ^+^CD24^+^ expression (**Figure 2F**), with a significant increase in their absolute numbers (**Figure 2G**). The accumulation of CD4^-^CD8^+^TCRβ^+^CD24^+^ cells in the thymus suggests the onset of T-ALL and migration of thymic tumor cells to the periphery such as spleen (40).

Similar to human pediatric T-ALL, radiation-induced murine T-ALL displayed elevated levels of *Notch1*, *Myc*, and *Lmo2* mRNAs, a hallmark of human T-ALL (**Supplementary Figure 3A**) (41, 42). Upregulation of those genes likely contributes to the transformation of normal thymic cells into T-ALL. Furthermore, radiation exposure led to the overexpression of pre-TCRα (*Ptcra*) mRNAs characteristic in proliferating DN to DP thymocytes, suggesting involvement of pre-TCRα signal in thymic hyperplasia (**Supplementary Figure 3B**). Sustained pre-TCRα expression may further enhance Notch1 signaling, playing a key role in T-ALL initiation and progression (43). Collectively, these results confirmed that radiation exposure reproducibly induces T-ALL in mice.

### 3.3 GCB-LMP1/2A mice exhibited a lower incidence of clonal lymphomas and a longer life span after irradiation

**Figure 3:**
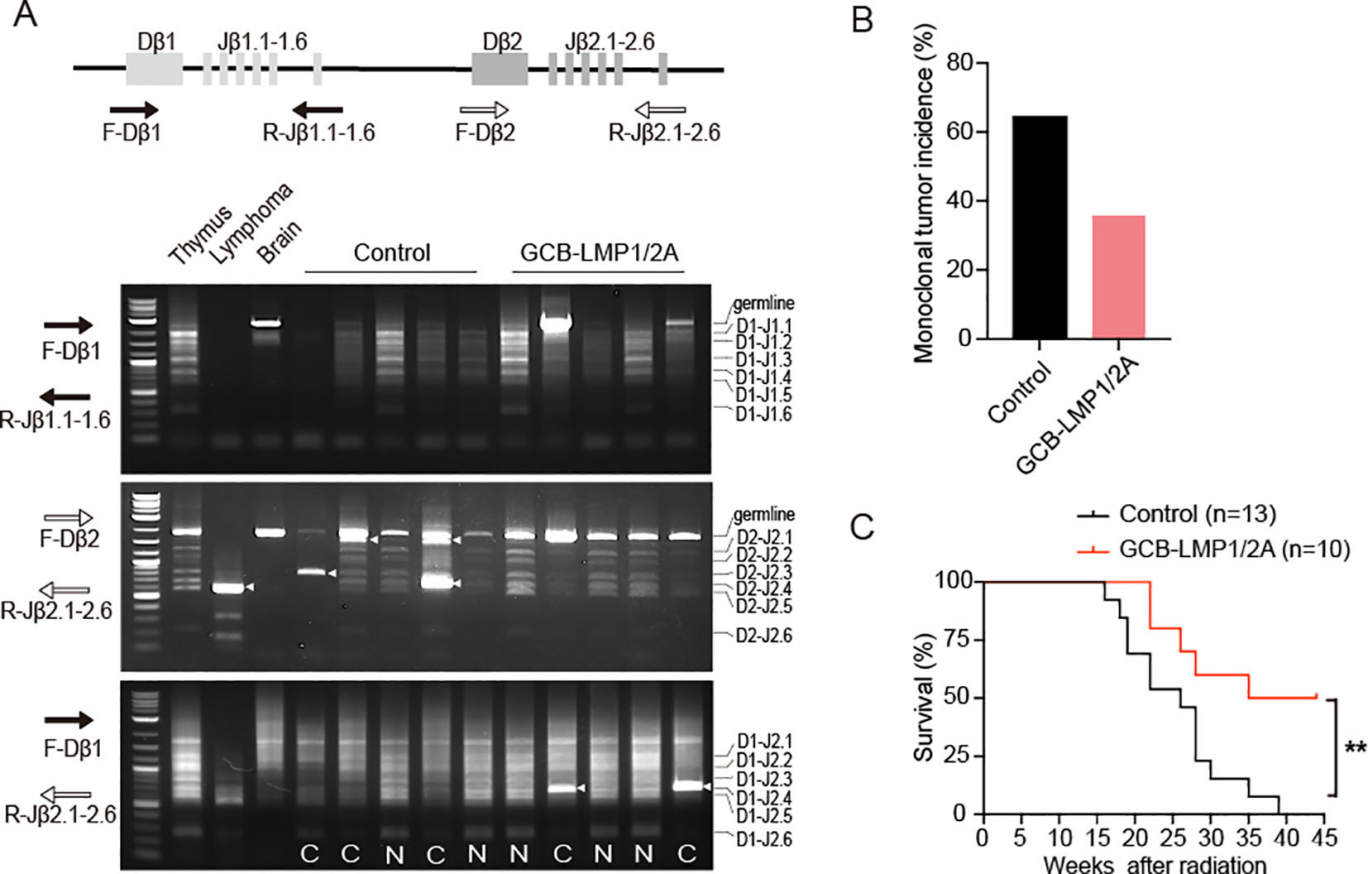
T-ALL incidence and survival curve of total body irradiated mice. **(A)** Illustration of the TCRβ locus and positions of the PCR primers used for detection of TCRβ rearrangements. The lower panels display gel electrophoresis of PCR products from three primer sets: F-Dβ 1/R-Jβ 1.6 (*top*), F-Dβ 2/Jβ 2.6 (*middle*), and F-Dβ 1/R-Jβ 2.6 (*bottom*). Clonal bands are indicated by arrowheads. N, Normal TCRβ rearrangements; C, Associated with clonal expansion. **(B)** Monoclonal tumor incidence of T-ALL, n=14 in each group. **(C)** Kaplan–Meier survival curves for mice after radiation (log-rank test, **p < 0.01). Number of mice are indicated.

Next, we evaluated the incidence of radiation-induced T-ALL. Following radiation exposure, clonal expansion occurs during the rearrangement of the TCRβ receptor locus, serving as one of the key early markers of T-ALL development. By assessing TCRβ rearrangement, we could determine the incidence of early tumor onset. We adopted PCR amplification to detect D-J recombination at TCRβ gene locus (31, 44, 45). Three sets of primers were used: one set for detecting recombination between the Dβ1 and Jβ1 loci, the second set for the Dβ2 and Jβ2 loci, and the third set for the Dβ1 and Jβ2 loci. DNA from the brain exhibited only a single germline band, serving as a control for the absence of recombination. DNA from the normal thymus contains polyclonal thymocytes, which exhibit multiple bands of uniform intensity due to recombination between various Dβ and Jβ loci. In contrast, DNA from the lymphoma exhibited single monoclonal band or a few oligoclonal bands with increased intensity. By assessing TCRβ rearrangements in thymocytes from control and GCB-LMP1/2A mice, we evaluated the incidence of clonal T-ALL expansion in thymus (**Figure 3A**). At 14-weeks post-radiation exposure, we examined both genotypes and found that the incidence of clonal T-ALL in GCB-LMP1/2A mice was 35.7%, significantly lower than the 64.3% observed in the control mice (**Figure 3B**). Furthermore, GCB-LMP1/2A mice exhibited a significantly longer lifespan following radiation exposure (**Figure 3C**). These data indicate that expression of EBV latency genes LMP1 and LMP2A in GC B cells activates host immune surveillance that reduces the incidence of radiation-induced T-ALL despite the absence of EBV antigens in T-ALL cells.

### 3.4 Effector and memory T cells induced by LMP1/2A^+^ B cells are maintained in GCB-LMP1/2A mice even after irradiation

**Figure 4:**
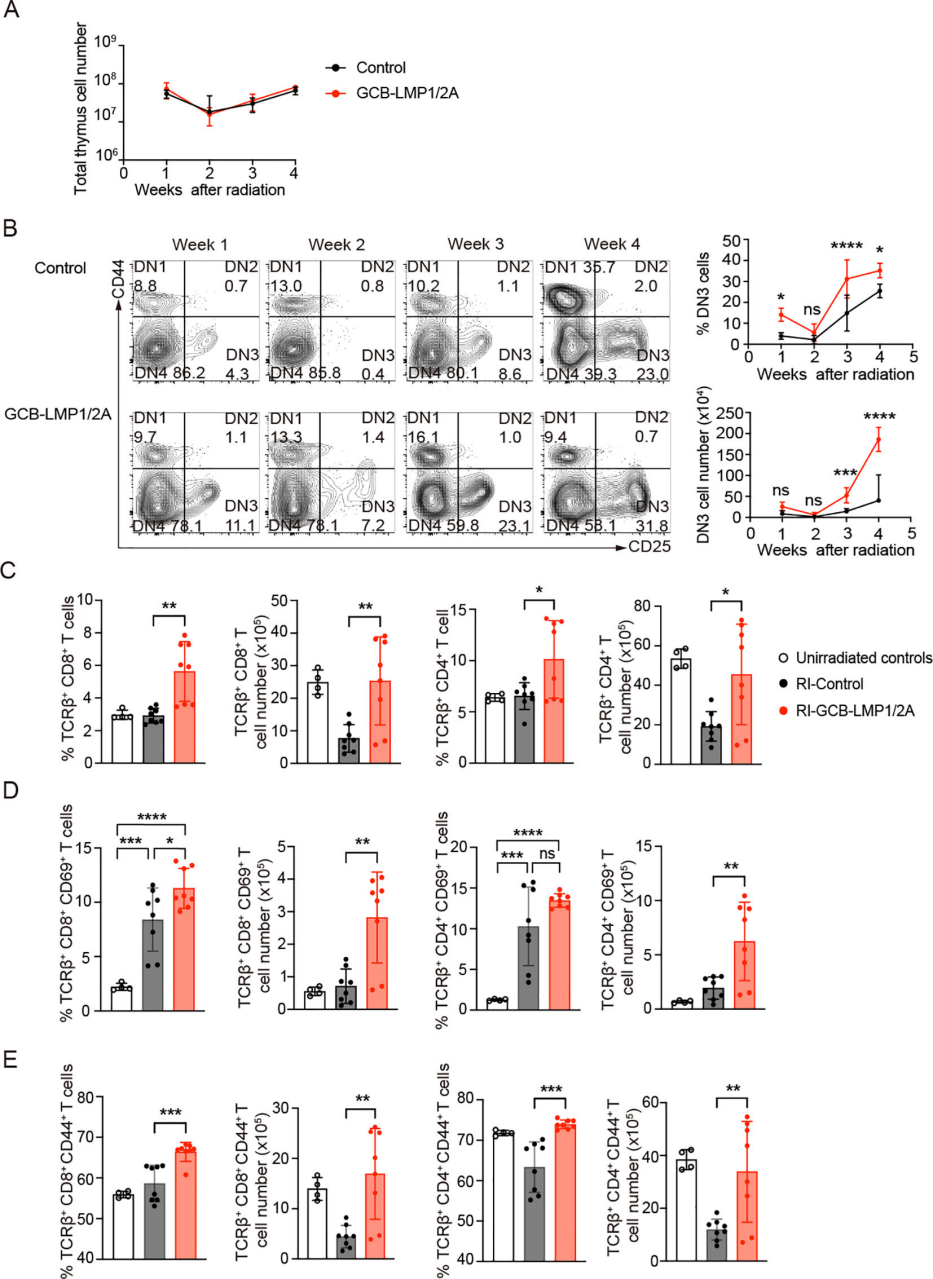
Changes in thymic cell populations following radiation exposure. **(A)** Total thymic cell counts after the last radiation exposure. Number of mice at week 1 (n=4), week 2 (n=5), week 3 (n=5), and week 4 (n=3). **(B)** Representative FACS plots of CD4 and CD8 double-negative cells in the thymus at indicate week after the last radiation (*left*). The percentage in DN fraction (*upper right*) and absolute number (*lower right*) of DN3 cells (CD44^-^CD25^+^) in thymus at indicated time points. Statistical analysis was performed using two-way ANOVA with Bonferroni’s multiple comparisons test; *p < 0.05; ***p < 0.001; ****p < 0.0001; ns, not significant. **(C-E)** Percentages and absolute numbers of each T cells in thymus at 3 weeks after the last radiation. **(C)** The percentage and absolute numbers of TCRβ^+^CD8^+^ and TCRβ^+^CD4^+^ T cells in thymus. **(D)** The Percentage and absolute numbers of CD69^+^ in TCRβ^+^CD8^+^ or TCRβ^+^CD4^+^ T cell fractions. **(E)** The Percentage and absolute numbers of CD44^+^ in TCRβ^+^CD8^+^ or TCRβ^+^CD4^+^ T cell fractions. Number of mice in unirradiated controls (n=4), RI-Control (n=8), and RI-GCB-LMP1/2A (n=8). Statistical analysis was performed using One-way ANOVA with Bonferroni’s multiple comparisons test; *p < 0.05; **p < 0.01; ***p < 0.001; ****p < 0.0001.

To uncover the underlying reasons for the lower incidence of T-ALL and a longer lifespan observed in GCB-LMP1/2A mice, we analyzed the acute phase of thymus within 4 weeks following radiation exposure. The thymic cell numbers decreased after radiation, especially the lowest levels occurring in the second week (**Figure 4A**). The FACS analysis revealed that reduction in DN3 stage was particularly lowest at week 2 and then turned to increase (**Figure 4B**). Existing studies have shown that when the competitive mechanisms of normal cells in the thymus are disrupted, thymic cells maintain a state of self-renewal, particularly DN3 stage cells, which could lead to the development of leukemia (46–48). Based on this, the reduced incidence of T-ALL in GCB-LMP1/2A mice can be explained by the impaired self-renewing DN3 cells in thymus. However, this seems unlikely because we observed a significantly higher number of DN3 cells in the thymus of GCB-LMP1/2A mice (**Figure 4B**). Another possible reason is the impaired replenishment of Lineage^-^Sca1^+^cKit^+^ (LSK) cells in the bone marrow (BM), a population of hematopoietic stem/progenitor cells (HSPCs) that can effectively resist radiation damage and improve the survival rates of mice with acute hematopoietic radiation syndrome (49–51). However, we observed no significant differences in the percentage of LSK cells in the BM (**Supplementary Figure 4A**). Therefore, these data suggest that the lower incidence of T-ALL in GCB-LMP1/2A mice is primarily influenced by environmental factors of T-ALL or its precursor cells within the thymus.

In the third week after the radiation exposure, where thymic cell numbers began to recover, we analyzed the state of mature T and NK cells in the thymus. GCB-LMP1/2A mice showed significantly higher percentages and numbers of mature αβ T cells in both CD4^+^ and CD8^+^ T cells compared to the control mice (**Figure 4C**). However, no significant increase was observed in NK cells or γδ T cells (**Supplementary Figures 4B, C**). Therefore, we hypothesized that the mature αβ T cells in the thymus may contribute to reduce the incidence of T-ALL in GCB-LMP1/2A mice. To test this hypothesis, we examined expression of activation markers of T cells, such as CD69 and PD-1. Radiation increased frequency of CD69, an early activation marker, on positive thymic CD4^+^ and CD8^+^ T cells compared to non-irradiated mice, where GCB-LMP1/2A mice showed significantly higher number of CD69^+^ activated CD4^+^ and CD8^+^ T cells compared to control mice (**Figure 4D**). Regarding the late activation marker PD-1, GCB-LMP1/2A mice showed significantly higher number of PD-1^+^ CD8^+^ T cells than control mice, whereas no significant increase was observed in cell number of PD-1^+^ CD4^+^ T cells between GCB-LMP1/2A mice and control mice (**Supplementary Figure 4D**).

Next, we evaluated status of memory T cells in GCB-LMP1/2A and control mice. CD44 is one of the most commonly used markers for memory T cells. We previously reported that expression of LMP1 and LMP2A in B cells rapidly upregulates CD44 upon T cell activation and its expression is maintained in memory T cells for more than 37 days (15). Frequency and cell number of CD44^+^ memory T cells displayed significantly higher levels in both CD4^+^ and CD8^+^ T cell fractions from GCB-LMP1/2A mice than control mice (**Figure 4E**). Additionally, GCB-LMP1/2A mice had more CD8^+^ T_EM_ cells in the BM compared to control mice (**Supplementary Figure 4E**). These findings suggest that memory T cells induced by LMP1/2A^+^ B cells are maintained in thymus and BM even after the radiation that potentially eliminate T-ALL and/or its precursor cells in thymus and other tissues. Such effects of T cell memory may also foster a microenvironment favoring thymic recovery, a process that may promote the proportion of DN3 cells, reduces the incidence of T-ALL, and extends the lifespan of GCB-LMP1/2A mice.

### 3.5 CD8^+^ T cells from GCB-LMP1/2A mice exhibit efficient killing of tumor cells

**Figure 5:**
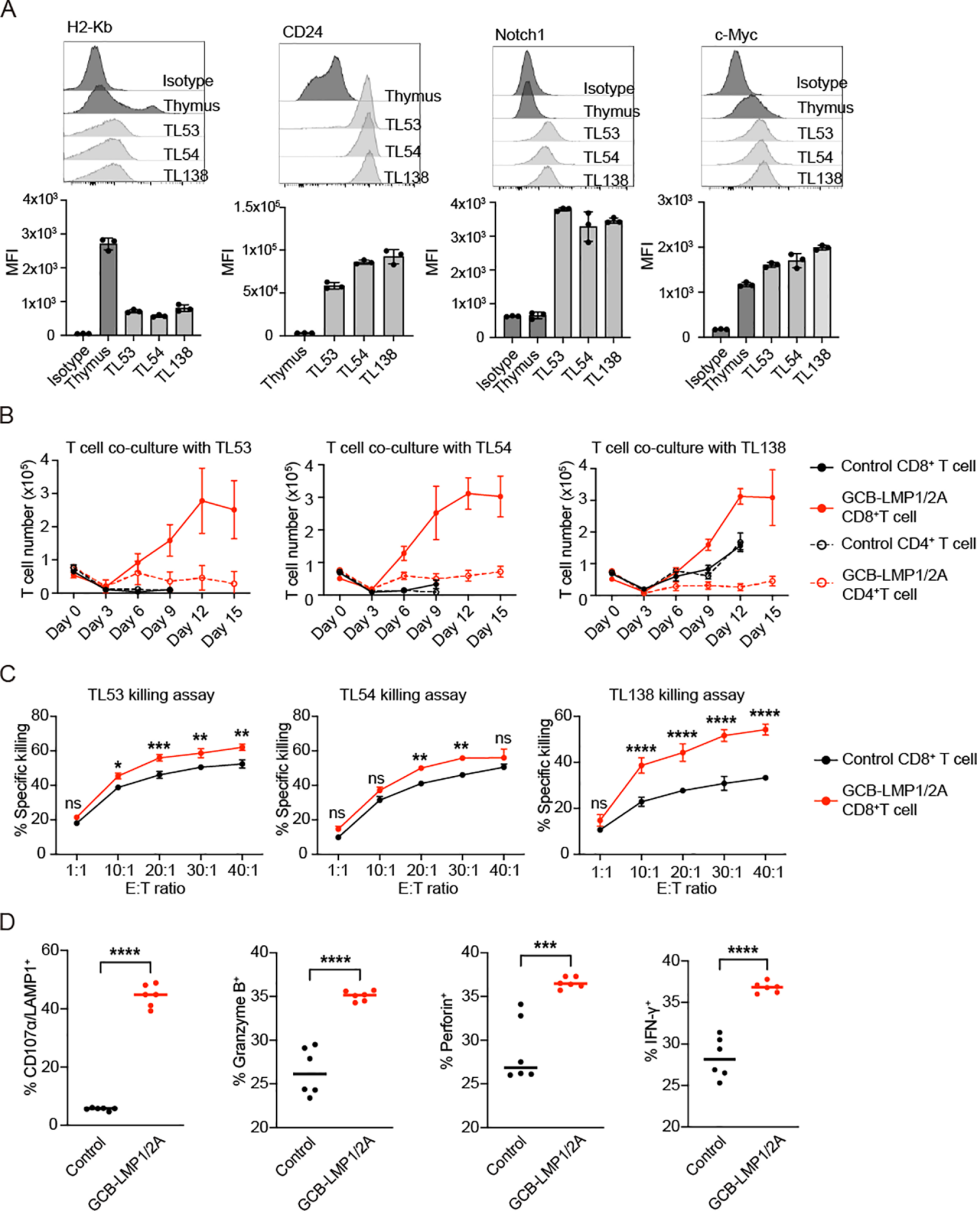
CD8^+^ T cells exhibit proliferation and cytotoxic activity after co-culture with tumor cells. **(A)** Flow cytometry of tumor cell lines (TL53, TL54, and TL138) assessed for H2-Kb, CD24, Notch1, and c-Myc expression. Histograms display fluorescence intensity profiles relative to normal thymus and isotype controls. Bar charts below each histogram represent mean fluorescence intensity (MFI) values. All tumor lines exhibit H2-Kb^-/low^, CD24^+^, Notch1^+^, and c-Myc^+^ phenotypes. **(B)** Co-culture of control and GCB-LMP1/2A T cells with tumor cell lines TL53, TL54, and TL138 over 15 days. CD8^+^ and CD4^+^ T cell number were counted every 3 days, with each point representing the mean ± SD from three replicates. **(C)** Killing assay of activated CD8^+^ T cells from control and GCB-LMP1/2A mice against tumor lines TL53, TL54, and TL138. CD8^+^ T cells were pre-activated *in vitro* by co-culture with B^LMP1/2A^ for 3 days. Killing efficiency was measured across different effector-to-target (E:T) ratios. Data represent the mean ± SD from three replicates. Statistical analysis was performed using two-way ANOVA with Bonferroni’s multiple comparisons test; *p < 0.05; **p < 0.01; ***p < 0.001; ****p < 0.0001; ns, not significant. **(D)** Percentages of CD107α/LAMP1^+^, Granzyme B^+^, Perforin^+^, and IFN-γ^+^ CD8^+^ T cells were measured after a 3-day co-culture of CD8^+^ T cells from the control and GCB-LMP1/2A (n=6 per group) with B^LMP1/2A^ cells. Statistical significance tested using an unpaired two-tailed Student’s t-test; ****p < 0.0001.

To assess the capacity of T cells to recognize and eliminate tumors, we selected terminal-stage T-ALL mice (over 20 weeks after the last radiation) and cultured thymic cells *in vitro*, establishing three stable T-ALL cell lines, named TL53, TL54, and TL138. TL53 and TL54 cell lines were established from control mice, whereas TL138 was from GCB-LMP1/2A mice. All three tumor cell lines exhibited downregulation of MHC class I (H2-Kb) and expressing CD24, Notch1, and c-Myc, typical markers for T-ALLs (**Figure 5A**). To evaluate T cell responses to tumor cells, CD4^+^ and CD8^+^ T cells were isolated from the spleens of control and GCB-LMP1/2A mice and co-cultured with each of three tumor cell lines in the presence of IL-2 to support proliferation. T cell number was determined every three days (**Figure 5B**). CD8^+^ T cells from GCB-LMP1/2A mice displayed robust proliferative responses to all tumor lines, rapidly expanding from day 3, peaking at day 12, and sustaining proliferation for more than 15 days. In contrast, CD8^+^ T cells from control mice showed minimal proliferation against the TL53 and TL54 cell lines and only a slight response to the TL138 cell line. CD4^+^ T cells displayed significantly lower proliferation, with limited response to TL53 and TL54, and only those from GCB-LMP1/2A mice showed modest proliferation in response to the TL138 cell line. The difference in proliferative capacity may be primarily due to CD8^+^ T cells from GCB-LMP1/2A mice being pre-activated *in vivo* by B^LMP1/2A^. As a result, these CD8^+^ T cells may not only exhibit a higher proportion of T_EM_ but also respond more rapidly to tumor cell stimulation compared to those from control mice.

To stimulate T cells by B^LMP1/2A^ cells, CD8^+^ T cells from GCB-LMP1/2A and control mice were co-cultured with B^LMP1/2A^ cells *in vitro* for three days. The activated CD8^+^ T cells were then isolated for tumor-killing assays to evaluate their cytotoxic capacity. Results showed that both groups of activated CD8^+^ T cells demonstrated tumor-killing activity, in particular, CD8^+^ T cells from GCB-LMP1/2A mice exhibited significantly higher killing efficiency (**Figure 5C**). Additionally, expression levels of CD107a/LAMP1, Granzyme B, Perforin, and IFN-γ were markedly upregulated in CD8^+^ T cells from GCB-LMP1/2A mice than in controls (**Figure 5D**). These results indicate that CD8^+^ T cells from GCB-LMP1/2A mice exhibit more potent proliferative and cytotoxic responses to T-ALL tumor cell lines, likely due to prior activation by B^LMP1/2A^ cells *in vivo*, enhancing their antitumor immune function.

**Figure 6:**
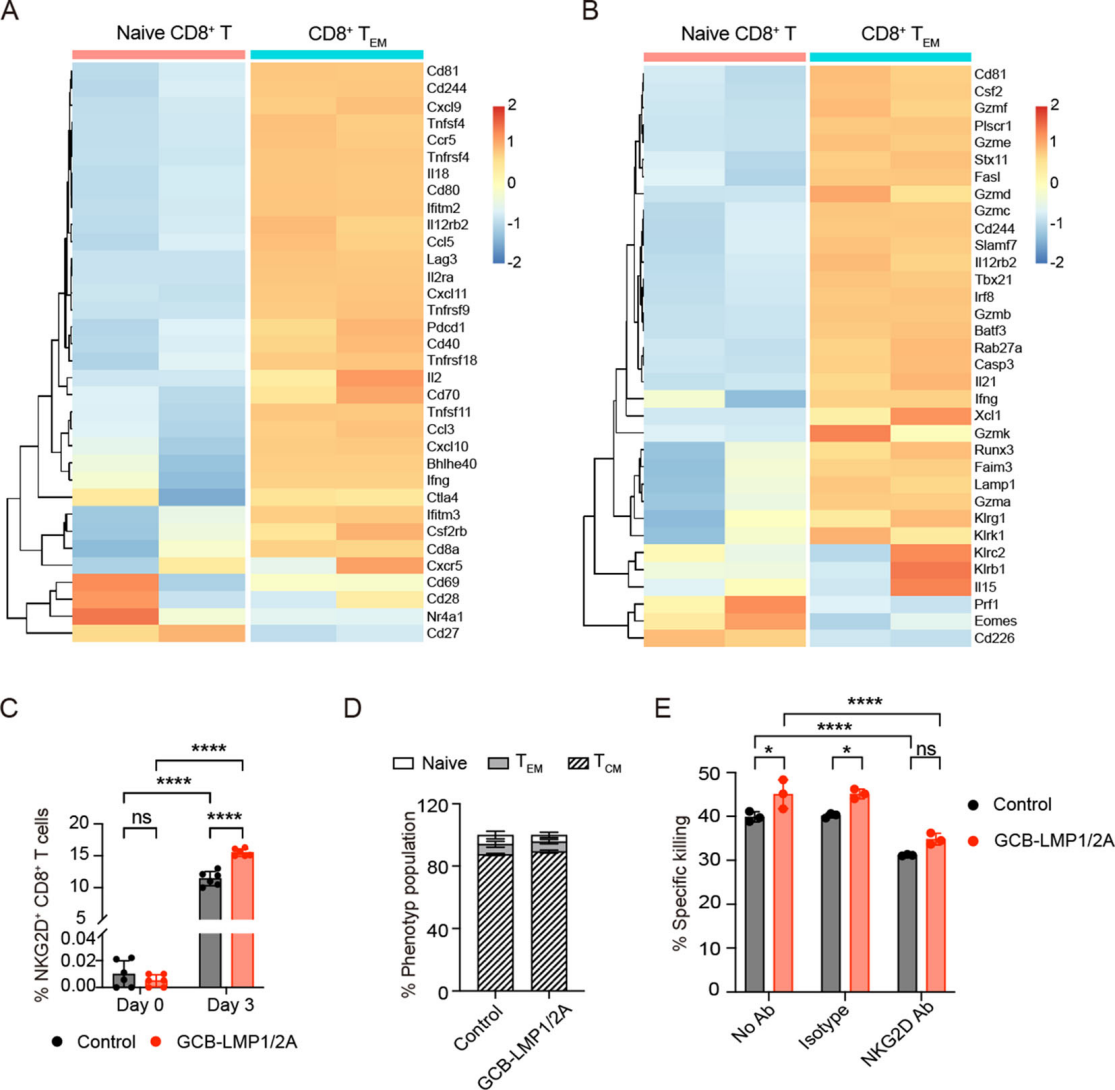
Differentially expressing genes in CD8^+^ T cells activated by LMP1/2A-expressing B cells. **(A, B)** CD8^+^ T_EM_ cells were purified on day 25 of *in vitro* culture with LMP1/2A-expressing tumor cells and analyzed for mRNA expression by microarray. Data were normalized by RMA normalization and log2 transformed. Heatmaps show the expression of activation-related genes **(A)** and cytotoxicity-related genes **(B)** in CD8^+^ T_EM_ cells compared to naive CD8^+^ T cells. **(C)** NKG2D expression levels on CD8^+^ T cells from GCB-LMP1/2A and control mice before and after co-culture with B^LMP1/2A^ cells on day 3. **(D)** Population of various phenotypes within the NKG2D^+^ CD8^+^ T cells. **(E)** Killing assay of TL53 cells with no antibody (No Ab), IgG1 isotype antibody (Isotype) and NKG2D antibody (NKG2D Ab). Data represent the mean ± SD from three replicates. Statistical analysis was performed using two-way ANOVA with Bonferroni’s multiple comparisons test; *p < 0.05; ****p < 0.0001; ns, not significant.

We characterized B^LMP1/2A^ cell-specific CD8^+^ T cells by isolating total RNA and performing microarray analysis to compare their transcriptomes between splenic naive CD8^+^ T cells and those CD8^+^ T_EM_ cells activated by LMP1/2A-expressing B-lymphoma cells (22, 24). Representative activation- and cytotoxicity-related genes were ranked by fold changes (**Figures 6A, B**). The analysis revealed that classical activation markers such as *Ifng*, *Il2*, and *Il18* were upregulated, indicating an enhanced immune response. The upregulation of chemokine genes *Ccl3*, *Ccl5*, *Cxcl9*, and *Cxcl10* suggests that these activated CD8^+^ T cells possess increased migratory capacity, allowing them to infiltrate the tumor microenvironment more effectively. Transcription factors such as *Tbx21*, *Runx3*, *Batf3*, and *Irf8* were also upregulated, indicating that these cells had differentiated into potent effector T cells with robust cytotoxic capabilities. Furthermore, the upregulation of multiple Granzyme genes highlights their remarkable cytotoxic potential. Notably, the expression levels of co-stimulatory receptors *Cd244* (2B4/SLAMF4), *Tnfrsf9* (4-1BB), *Tnfrsf18* (GITR), *Tnfrsf4* (OX40), *Cd80*, and *Cd70*, along with NK cell-related genes *Klrk1* (NKG2D) and *Klrc2* (NKG2C), were significantly increased. These molecules provide additional activation signals, enhancing the ability of CD8^+^ T cells to recognize and eliminate tumor cells with reduced or absent MHC class I expression, thereby reinforcing immune surveillance.

Next, we performed qPCR analysis to validate the mRNA expression levels of the cell surface receptors *Klrk1* (NKG2D) and *Cd244* (2B4/SLAMF4), which are associated with CD8^+^ T cell activation and cytotoxicity. The results showed that after stimulation with B^LMP1/2A^ cells or T-ALL tumor cells, the expression of *Klrk1* and *Cd244* was significantly higher in CD8^+^ T cells from GCB-LMP1/2A mice compared to the control group (**Supplementary Figures 5A, B**). To further evaluate the roles of these genes in tumor recognition and immune response, we examined the expression levels of their corresponding ligands, *Ulbp1* (ligand for NKG2D) and *Cd48* (ligand for 2B4/SLAMF4), on B^LMP1/2A^ cells and T-ALL tumor cells. The results indicated that both ligands were highly expressed in these cells (**Supplementary Figures 5C, D**).

We then measured the protein expression of NKG2D and 2B4 on the surface of CD8^+^ T cells using specific monoclonal antibodies. After co-culturing CD8^+^ T cells from both the control and GCB-LMP1/2A groups with B^LMP1/2A^ cells for 3 days, we detected substantial NKG2D^+^ (**Figure 6C**) and 2B4^+^ cells (**Supplementary Figure 5E**). Notably, CD8^+^ T cells from GCB-LMP1/2A mice exhibited significantly higher expression of NKG2D and 2B4 compared to the control group, consistent with the qPCR results. Further analysis showed that the majority of NKG2D^+^ and 2B4^+^ CD8^+^ T cells were of the T_CM_ phenotype (**Figure 6D**, **Supplementary Figure 5F**).

Next, we investigated whether NKG2D and 2B4 play important role in CD8^+^ T cell-mediated killing of tumor cells. Blocking of the NKG2D receptor on CD8^+^ T cells led to a moderate but statistically significant reduction in their cytotoxic activity against TL53 cells, compared with no antibody or isotype control conditions. This suggests that the enhanced killing ability of CD8^+^ T cells from GCB-LMP1/2A mice is at least partially mediated by NKG2D–NKG2D ligand (NKG2DL) interactions (**Figure 6E**). However, the incomplete loss of cytotoxicity upon NKG2D blocking indicates that additional mechanisms, such as classical MHC I–mediated recognition or other co-activation pathways, may also contribute to tumor cell elimination. In contrast, blocking of the 2B4 receptor did not lead to a measurable reduction in killing efficiency in this system, suggesting that the 2B4–CD48 interaction may not play a dominant role under these experimental conditions. (**Supplementary Figure 5G**).

### 3.6 LMP1/2A-induced immune surveillance suppresses intestinal tumor development

**Figure 7:**
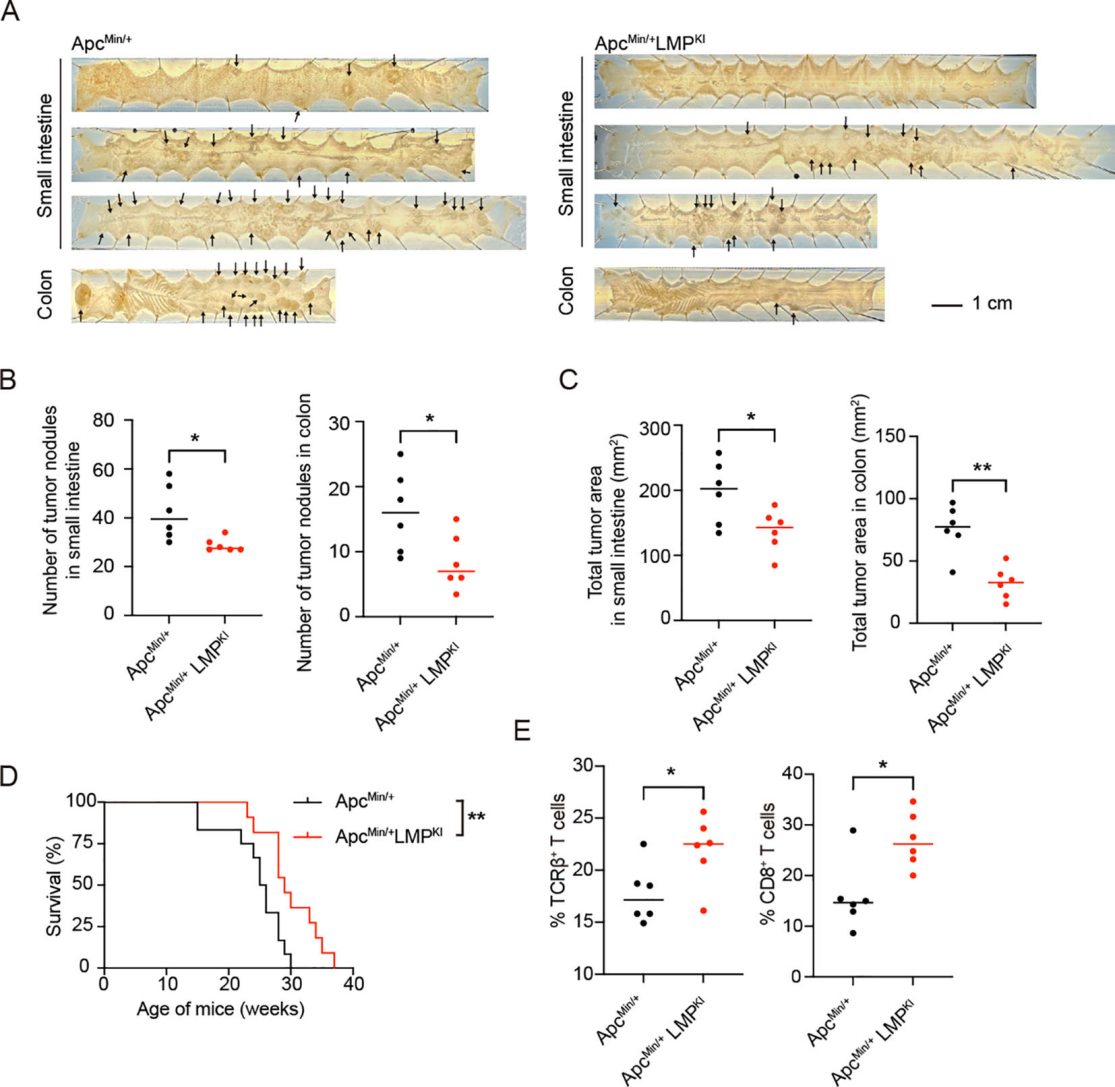
Analysis of LMP1/2A-induced immune surveillance in the Apc^Min^-induced intestinal tumor formation. **(A)** Representative macroscopic images of small intestines and colons from Apc^Min/+^ and Apc^Min/+^LMP^KI^ mice at 22-week-old with arrows indicating tumor nodules. Scale bar=1 cm. **(B, C)** Quantification of tumor nodules number in small intestine and colon **(B)**. Quantification of total tumor area in small intestine and colon **(C)**. Each dot represents an individual mouse (n=6 per group). Statistical analysis was performed using an unpaired two-tailed Student’s *t* test; *p < 0.05; **p < 0.01. **(D)** Kaplan-Meier survival curves for Apc^Min/+^ and Apc^Min/+^LMP^KI^ mice (n=12 per group; log-rank test, **p < 0.01). **(E)** Flow cytometry analysis of tumor-infiltrating lymphocytes (TILs), showing percentages of TCRβ^+^ T cells in CD45^+^ lymphocytes fractions and CD8^+^ in TCRβ^+^ T cell fractions in Apc^Min/+^ and Apc^Min/+^LMP^KI^ mice (n=6 per group). Statistical analysis was performed using an unpaired two-tailed Student’s *t* test; *p < 0.05.

Given the demonstrated suppressing effect of EBV-induced immune surveillance to T-ALL, we further explored its potential effects on other solid tumors. We utilized the Apc^Min/+^ mouse model, which harbors a mutation in the *Apc* gene and is prone to developing intestinal tumor (52). By crossing Apc^Min/+^ mice with GCB-LMP1/2A mice, we generated mice carrying both LMP1/2A antigens and Apc^Min/+^ mutation (referred to as Apc^Min/+^LMP^KI^) to assess whether LMP1/2A-induced immune surveillance could suppress intestinal tumor development. At 22 weeks of age, significant intestinal tumor formation was observed in these mice. Surprisingly, the tumor burden of Apc^Min/+^LMP^KI^ mice was lower compared to Apc^Min/+^ mice (**Figure 7A**). The number of tumor nodules (**Figure 7B**) and total tumor areas (**Figure 7C**) were significantly reduced in Apc^Min/+^LMP^KI^ mice compared to Apc^Min/+^ controls. As predicted by the lower tumor burden, survival period was extended in Apc^Min/+^LMP^KI^ mice compared to Apc^Min/+^ controls (**Figure 7D**).

To investigate the underlying mechanisms, we isolated intra-tumoral lymphocytes and found a higher proportion of αβ T cells infiltrating the tumors of Apc^Min/+^LMP^KI^ mice, particularly an increased percentage of CD8^+^ T cells (**Figure 7E**). These findings indicate that LMP1/2A-induced immune surveillance enhances both the intra-tumoral infiltration and activation of CD8^+^ T cells. Thus, beyond its resistance to T-ALL, LMP1/2A-induced immune surveillance also promotes anti-tumor T cell responses in the solid tumor model.

Taken together, our study uncovered a potential mechanism by which LMP1/2A-expression in B cells enhances CD8^+^ T cell-mediated immune responses to combat both hematological malignancies and solid tumors. These findings highlight beneficial aspects of EBV infection in humans that may contribute to the surveillance of pan-tumor development, providing an important insight into cancer prevention.

## 4 Discussion

In recent years, growing evidence has shown that viral infections can enhance host immune defense not only through antigen-specific responses but also by activating bystander T cells, promoting cross-presentation, and sustaining innate immune signaling (53). This phenomenon, often referred to as “virus-induced cross-protection (54)” has been demonstrated in various models. For example, latent infection with murine gammaherpesvirus MHV68 has been shown to alleviate allergic asthma (3) and enhance resistance to bacterial infections (2), likely through prolonged innate immune activation. Similarly, EBV, a human-specific gammaherpesvirus, may shape host immunity through comparable mechanisms, as co-infection with EBV and CMV has been reported to expand memory-like NKG2C^+^ NK cells (55), suggesting its potential role in enhancing immune surveillance. Unlike these studies that primarily focus on innate immunity, our study demonstrates that the EBV latent genes LMP1 and LMP2A, through the transformation of B cells, enhance the expression of stress-induced antigens such as NKG2D ligands and promote the development of a population of CD8^+^ T cells with broad responsiveness. These T cells are capable of recognizing not only viral antigens but also stress signals, thereby contributing to immune surveillance against non-viral tumors.

In our model, LMP1/2A expression in GC B cells effectively suppressed the development of various EBV-negative cancers through CD8^+^ T cell-mediated immune surveillance. This complements previous studies on the role of LMP1 in activating CD4^+^ and CD8^+^ CTLs, which primarily focused on their responses to LMP1/2A expressing B cell lymphomas (12, 15, 16). Here, we show that LMP1/2A expression in GC B cells leads to robust CD8^+^ T cell activation, likely in gut-associated lymphoid tissues where GC reactions occur. These activated T cells may differentiate into effector and central memory subsets and exert systemic immune surveillance through peripheral circulation.

Most studies on radiation-induced T cell lymphomas have focused on oncogenic mechanisms, with relatively little focus on tumor clearance. Our findings demonstrated that immune surveillance induced by LMP1/2A expression significantly reduced the incidence of early-stage monoclonal T-ALL development and extended survival after radiation exposure. Although the percentage of hematopoietic stem and progenitor cells, a source of T-ALLs, was not disturbed, GCB-LMP1/2A mice had significantly higher percentages of CD8^+^ memory T cells in the bone marrow and thymus compared with control mice, suggesting that CD8^+^ T cell-mediated immune surveillance plays an important role in the suppression of T-ALL.

Further analysis revealed that LMP1/2A-activated CD8^+^ T cells upregulated various activation and cytotoxicity-related molecules, including IFN-γ, Granzyme B, and Perforin, which likely enhanced their cytotoxic function. Additionally, the upregulation of chemokine genes such as *CXCL9* and *CXCL10* may facilitate T cell migration to the tumor microenvironment, further strengthening their immune surveillance capabilities. Notably, when T-ALL cells were cultured *in vitro* and transformed into MHC class I-low/negative tumor cells, LMP1/2A-activated CD8^+^ T cells still demonstrated effective cytotoxicity against these tumor cells. This suggests that, beyond the MHC class I-mediated pathway, other mechanisms contribute to the activation and effector functions of these T cells.

This study revealed that 2B4/SLAMF4 and NKG2D, key receptors known for regulating NK cell functions, were also significantly upregulated CD8^+^ T cells activated by LMP1/2A^+^B cells. Their corresponding ligands, Ulbp1 and CD48, were highly expressed on the tumor cell surface, providing crucial activation signals for CD8^+^ T cell recognition of MHC class I-low or -negative tumors. In *in vitro* killing assays, blocking NKG2D moderately reduced cytotoxicity against tumors with high Ulbp1 and extremely low MHC class I expression, suggesting a functional role of this axis under such specific conditions. However, the killing efficiency was not complete and inconsistently reproduced in two additional tumor lines with lower NKG2DL or higher MHC calss I expression. These results indicate that additional mechanisms—such as classical MHC I–dependent recognition or other co-stimulatory pathways—may also contribute to CD8^+^ T cell-mediated tumor elimination. We thus consider NKG2D–NKG2DL interactions to be one of several pathways involved in this process, but not the only mechanism. Conversely, blocking 2B4/SLAMF4 did not significantly impair killing activity. However, the blocking efficiency of the anti-2B4 antibody used has not been verified in our system, and its potential role may be compensated by alternative cytotoxic pathways. Therefore, we cannot rule out a functional contribution of the 2B4–CD48 axis, although it does not appear to play a dominant role under the current experimental conditions.

While NKG2DLs are stress-induced molecules not typically expressed in normal tissues, many types of tumors express these ligands, enabling NKG2D^+^ lymphocytes to initiate potent immune responses via cytokine secretion and cytotoxic effector functions (52, 56, 57). The presence of NKG2DLs in tumors, along with evidence from *in vitro* assays and mouse models, has firmly established the role of the NKG2D–NKG2DL axis in antitumor immunity (58–61). Human studies have further demonstrated that NKG2DLs are upregulated during the lytic phase of EBV infection, enhancing the susceptibility of infected cells to NKG2D^+^ NK and T cell–mediated clearance (62, 63). Additionally, NKG2D CAR-T cells have shown therapeutic efficacy not only in EBV-associated lymphomas (64) but also in human T-ALL (65), indicating that NKG2D-mediated immune recognition may provide a broadly applicable mechanism for tumor control in both virus-related and unrelated malignancies.

Our study extends this relevance to radiation-induced T-ALL, showing that CD8^+^ T cells activated by LMP1/2A^+^B cells exhibit increased NKG2D expression, which enhances their ability to recognize and eliminate tumors lacking MHC class I. In future studies, we plan to use MHC class I-deficient tumor models or MHC class I–neutralizing antibodies to completely block the classical killing pathway, in order to further evaluate the roles of NKG2D, 2B4, and other potential mechanisms. If the MHC I–dependent mechanism remains dominant, we also intend to explore candidate TAAs, aiming for a more comprehensive understanding of how LMP1/2A^+^B cell–induced CD8^+^ T cells recognize and eliminate tumors.

In addition to hematological malignancies, LMP1/2A-induced immune surveillance also exhibited anti-tumor effects in intestinal tumor model. By crossing GCB-LMP1/2A mice with Apc^Min/+^ mice, we found that LMP1/2A-induced immune surveillance significantly reduced the tumor burden of intestinal tumor in Apc^Min/+^ mice and extended their survival. Further analysis indicated that LMP1/2A likely enhances the infiltration efficiency and activation of CD8^+^ T cells, promoting anti-tumor immune responses within the tumor microenvironment. These findings suggest that LMP1/2A plays a critical role not only in hematological malignancies but also demonstrates potential anti-tumor capabilities in solid tumors.

This study highlights the critical role of LMP1/2A in suppressing hematological and solid tumors by enhancing CD8^+^ T cell-mediated immune surveillance, providing a basis for the development of novel therapeutic and preventive strategies targeting MHC class I-negative malignancies across a broad range of cancer types.

Given its strong immunostimulatory properties, LMP1/2A may serve as a molecular tool in future immunotherapy or cancer vaccine strategies. However, its oncogenic potential and ability to modulate the tumor microenvironment underscore the need for careful evaluation of safety and context-specific effects before clinical application. Additionally, although our study highlights the central role of CD8^+^ T cells in LMP1/2A-induced tumor immune surveillance, we cannot exclude the potential contribution of NK cells or NKT cells. Given the observed activation of the NKG2D pathway, these innate lymphocyte subsets may also exhibit enhanced cytotoxic function through recognition of stress-induced ligands. While we did not observe significant differences in NK cell numbers between tumor-bearing and protected mice, we did not directly assess the functional status of NK or NKT cells, such as activation marker expression, cytokine production, or granule-mediated cytotoxicity. Therefore, their role in the observed immune protection remains unclear. Future studies should investigate the activation and effector profiles of NK and NKT cells to better understand the potential interplay between innate and adaptive immunity in the context of LMP1/2A-mediated tumor control.

## Data Availability

The original contributions presented in the study are included in the article/**Supplementary Material**. Further inquiries can be directed to the corresponding author.

## Supplementary material

Supplementary Figure 1 Generation of knock-in mice conditionally expressing LMP1 and LMP2A in a Cre-dependent manner. **(A)** Strategy for targeting conditional LMP1 and LMP2A alleles into the Rosa26 locus. **(B)** Western blot analysis of LMP1 and LMP2A expression in *in vitro*-expanded B cells from GCB-LMP1/2A mice (B^LMP1/2A^). CD40-activated B cells (CD40 act-B) from control mice were used as negative controls. β-Actin was used as a loading control. **(C)** Percentage of GC B cells in the PPs and mLNs of GCB-LMP1/2A mice compared to control mice. Each dot represents an individual mouse (n=4). Statistical significance tested using an unpaired two-tailed Student’s t-test; ***p* < 0.01; ****p* < 0.001. **(D)** Representative of FACS results of CD8^+^ T cells gated for effector memory (T_EM_), central memory (T_CM_), and naive subsets based on CD44 and CD62L expression at the indicated time points during co-culture with either CD40 act-B cells (T+CD40 act-B) or B^LMP1/2A^ cells (T+B^LMP1/2A^). **(E)** CD8^+^ T_EM_ cell numbers were quantified at the indicated time points.

Supplementary Figure 2 Post-irradiation thymic and splenic hyperplasia exhibits an immature T cell phenotype. **(A)** Total cell numbers in the thymus and spleen of non-irradiated control mice (NC) and mice after 15–30 weeks of radiation (RI). Statistical analysis was performed using multiple t-tests, with Holm-Šidák correction for multiple comparisons; ***p* < 0.01. **(B)** Representative FACS analysis showing the percentage of CD24^+^CD8^+^ immature T cells in the spleen. **(C)** Representative FACS analysis displaying the percentage of CD19^+^ B cells and CD3^+^ T cells in the spleen.

Supplementary Figure 3 Expression analysis of T-ALL specific genes. **(A, B)** Quantitative RT-PCR analysis of sorted thymic cell subsets, including DN (CD4^-^CD8^-^), DP (CD4^+^CD8^+^), 4SP (CD4^+^CD8^-^), and 8SP (CD4^-^CD8^+^), from mice 14 weeks post-radiation exposure compared to control mice that did not undergo radiation. The expression levels of *Notch1*, *Myc*, and *Lmo2* are presented in **(A)**. The expression levels of pre-TCRα (*Ptcra*) presented in **(B)**. Data from three technical replicates is shown. Statistical analysis was performed using multiple t-tests, with Holm-Šidák correction for multiple comparisons; ***p* < 0.01; ****p* < 0.001; *****p* < 0.0001; ns, not significant.

Supplementary Figure 4 Changes in various cell populations in bone marrow and thymus 3 weeks after the last radiation dose. **(A)** Percentage of LSK cells (Lineage^-^Sca1^+^cKit^+^) in bone marrow (BM). **(B)** Percentages and absolute numbers of NK cells in thymus. **(C)** Percentages and absolute numbers of γδ T cells in thymus. **(D)** The Percentage and absolute numbers of PD-1^+^ in TCRβ^+^CD8^+^ or TCRβ^+^CD4^+^ T cell fractions in the thymus. **(E)** Percentage of CD8^+^ T_EM_ and CD4^+^ T_EM_ cells in BM. Statistical significance tested using one-way ANOVA with Bonferroni’s multiple comparisons test; **p* < 0.05; ***p* < 0.01; *****p* < 0.0001; ns, not significant.

Supplementary Figure 5 Expression of cytotoxicity related genes in CD8^+^ T cells and tumor cells and their impact on cytotoxic function. Relative mRNA expression levels of *Klrk1* **(A)** and *Cd244* **(B)** in CD8^+^ T cells before stimulation and after 3 days of stimulation with B^LMP1/2A^ cells or tumor cells. Data from three technical replicates is shown. Statistical analysis was performed using multiple t-tests, with Holm-Šidák correction for multiple comparisons; **p* < 0.05; ***p* < 0.01; ****p* < 0.001; ns, not significant. **(C, D)** Relative mRNA expression levels of *Ulbp1* **(C)** and *Cd48* **(D)** in normal B cells, B^LMP1/2A^ cells, normal DP cells, and tumor cells. **(E)** 2B4 expression levels on CD8^+^ T cells from GCB-LMP1/2A and control mice after co-culture with B^LMP1/2A^ cells on day 3. **(F)** Population of various phenotypes within the 2B4 ^+^ CD8^+^ T cells. **(G)** Killing assay of TL53 cells with no antibody (No Ab), IgG2a isotype antibody (Isotype) and 2B4 antibody (2B4 Ab). Data represent the mean ± SD from three replicates. Statistical analysis was performed using two-way ANOVA with Bonferroni’s multiple comparisons test and unpaired two-tailed Student’s t-test; **p* < 0.05; ***p* < 0.01; ****p* < 0.001; *****p* < 0.0001; ns, not significant.
