# Supplementary material for: T cell-mediated immune surveillance conferred by latent Epstein-Barr virus genes suppresses a broad spectrum of tumor formation through NKG2D-NKG2DL interactions
Source: Front Immunol. 2025 Jun 4;16:1597731. doi: 10.3389/fimmu.2025.1597731 (PMC12174148; doi:10.3389/fimmu.2025.1597731)
Supplement: Supplementary Figure 1 — Generation of knock-in mice conditionally expressing LMP1 and LMP2A in a Cre-dependent manner. (A) Strategy for targeting conditional LMP1 and LMP2A alleles into the Rosa26 locus. (B) Western blot analysis of LMP1 and LMP2A expression in in vitro-expanded B cells from GCB-LMP1/2A mice (BLMP1/2A). CD40-activated B cells (CD40 act-B) from control mice were used as negative controls. β-Actin was used as a loading control. (C) Percentage of GC B cells in the PPs and mLNs of GCB-LMP1/2A mice compared to control mice. Each dot represents an individual mouse (n=4). Statistical significance tested using an unpaired two-tailed Student’s t-test; **p < 0.01; ***p < 0.001. (D) Representative of FACS results of CD8+ T cells gated for effector memory (TEM), central memory (TCM), and naive subsets based on CD44 and CD62L expression at the indicated time points during co-culture with either CD40 act-B cells (T+CD40 act-B) or BLMP1/2A cells (T+BLMP1/2A). (E) CD8+ TEM cell numbers were quantified at the indicated time points. [file Image1.pdf]

Supplementary Material

1 Supplementary Data

Supplementary Material include 5 supplementary figures.

2 Supplementary Figures and Tables

2.1 Supplementary Figures

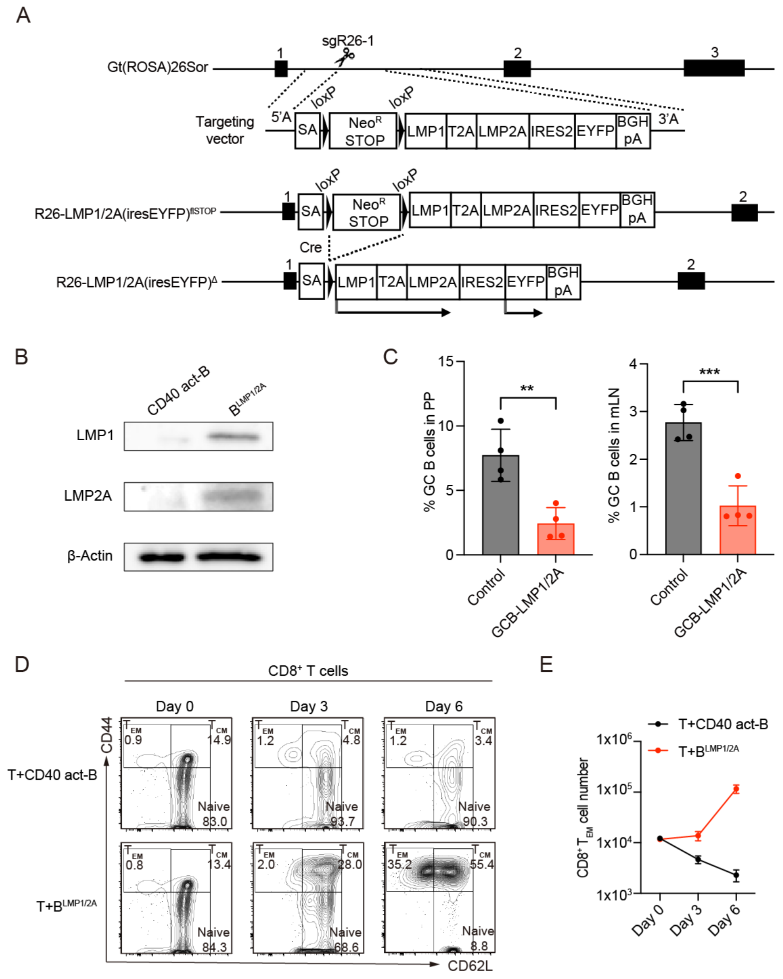

Supplementary Figure 1. Generation of knock-in mice conditionally expressing LMP1 and LMP2A in a Cre-dependent manner.

(A) Strategy for targeting conditional LMP1 and LMP2A alleles into the Rosa26 locus. **(B)** Western blot analysis of LMP1 and LMP2A expression in *in vitro*-expanded B cells from GCB-LMP1/2A mice ($B^{LMP1/2A}$). CD40-activated B cells (CD40 act-B) from control mice were used as negative controls. β-Actin was used as a loading control. **(C)** Percentage of GC B cells in the PPs and mLNs of GCB-LMP1/2A mice compared to control mice. Each dot represents an individual mouse (n=4). Statistical significance tested using an unpaired two-tailed Student's t-test; $**p < 0.01$; $***p < 0.001$. **(D)** Representative of FACS results of $CD8^+$ T cells gated for effector memory (T_{EM}), central memory (T_{CM}), and naive subsets based on CD44 and CD62L expression at the indicated time points during co-culture with either CD40 act-B cells (T+CD40 act-B) or $B^{LMP1/2A}$ cells ($T+B^{LMP1/2A}$). **(E)** $CD8^+$ T_{EM} cell numbers were quantified at the indicated time points.

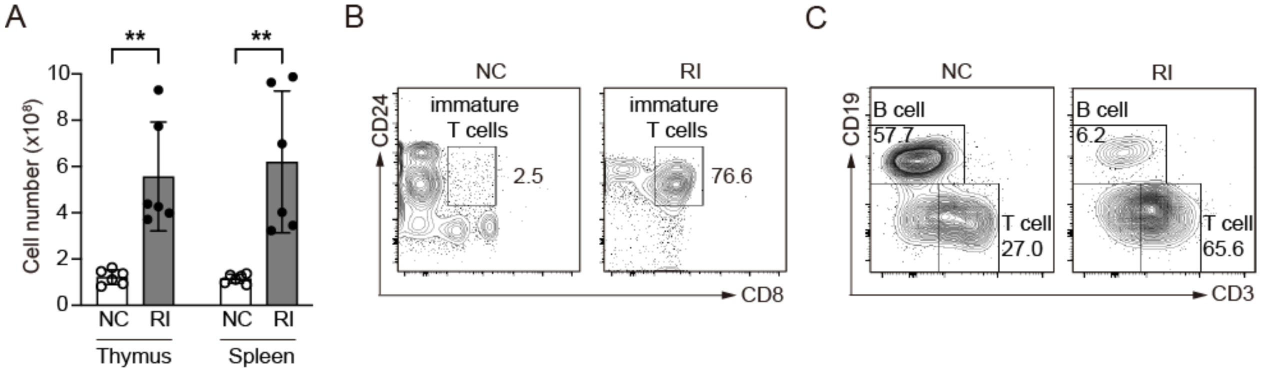

Supplementary Figure 2. Post-irradiation thymic and splenic hyperplasia exhibits an immature T cell phenotype.

(A) Total cell numbers in the thymus and spleen of non-irradiated control mice (NC) and mice after 15-30 weeks of radiation (RI). Statistical analysis was performed using multiple t-tests, with Holm-Šidák correction for multiple comparisons; $^{**}p < 0.01$. **(B)** Representative FACS analysis showing the percentage of $CD24^+CD8^+$ immature T cells in the spleen. **(C)** Representative FACS analysis displaying the percentage of $CD19^+$ B cells and $CD3^+$ T cells in the spleen.

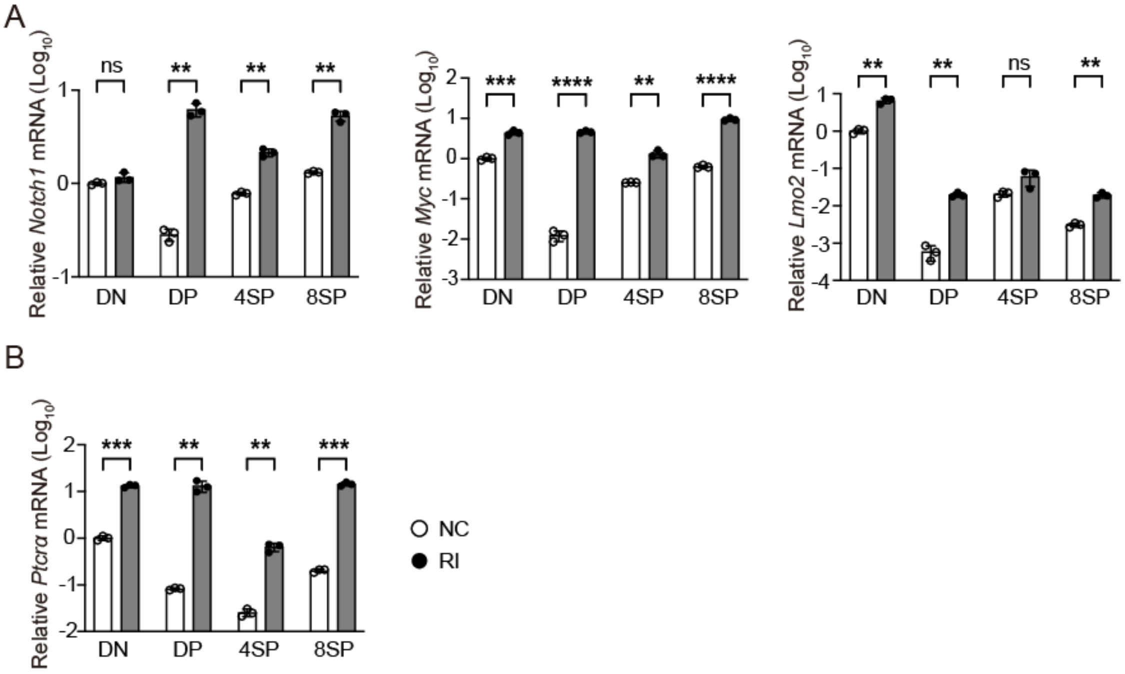

Supplementary Figure 3. Expression analysis of T-ALL specific genes.

(A, B) Quantitative RT-PCR analysis of sorted thymic cell subsets, including DN ($CD4^-CD8^-$), DP ($CD4^+CD8^+$), 4SP ($CD4^+CD8^-$), and 8SP ($CD4^-CD8^+$), from mice 14 weeks post-radiation exposure compared to control mice that did not undergo radiation. The expression levels of *Notch1*, *Myc*, and *Lmo2* are presented in **(A)**. The expression levels of pre-TCRα (*Ptcra*) presented in **(B)**. Data from three technical replicates is shown. Statistical analysis was performed using multiple t-tests, with Holm-Šidák correction for multiple comparisons; $^{**}p < 0.01$; $^{***}p < 0.001$; $^{****}p < 0.0001$; ns, not significant.

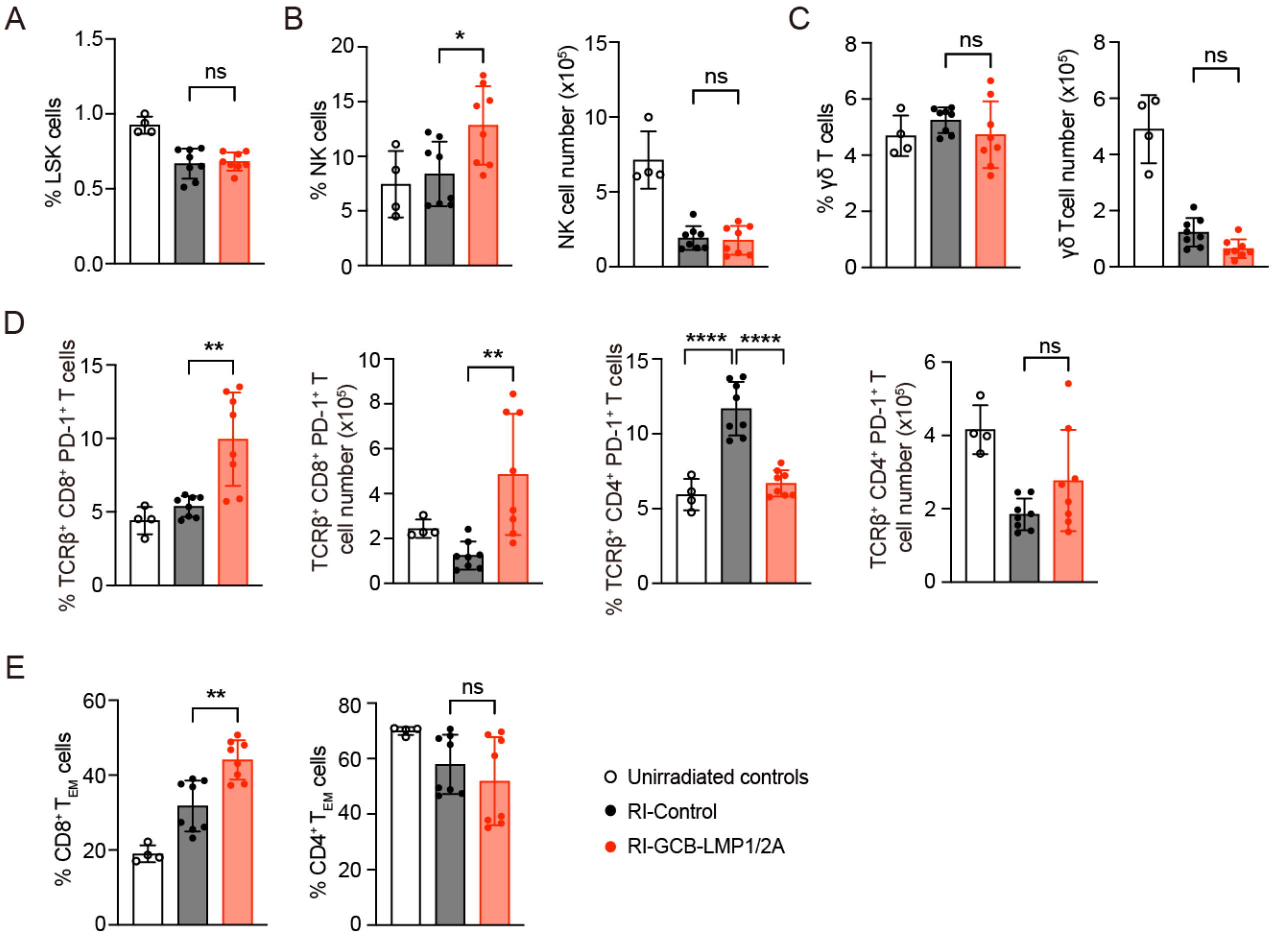

Supplementary Figure 4. Changes in various cell populations in bone marrow and thymus 3 weeks after the last radiation dose.

(A) Percentage of LSK cells (Lineage⁻Sca1⁺cKit⁺) in bone marrow (BM). **(B)** Percentages and absolute numbers of NK cells in thymus. **(C)** Percentages and absolute numbers of γδ T cells in thymus. **(D)** The Percentage and absolute numbers of PD-1⁺ in TCRβ⁺CD8⁺ or TCRβ⁺CD4⁺ T cell fractions in the thymus. **(E)** Percentage of CD8⁺ T_{EM} and CD4⁺ T_{EM} cells in BM. Statistical significance tested using one-way ANOVA with Bonferroni's multiple comparisons test; *$p < 0.05$; **$p < 0.01$; ****$p < 0.0001$; ns, not significant.

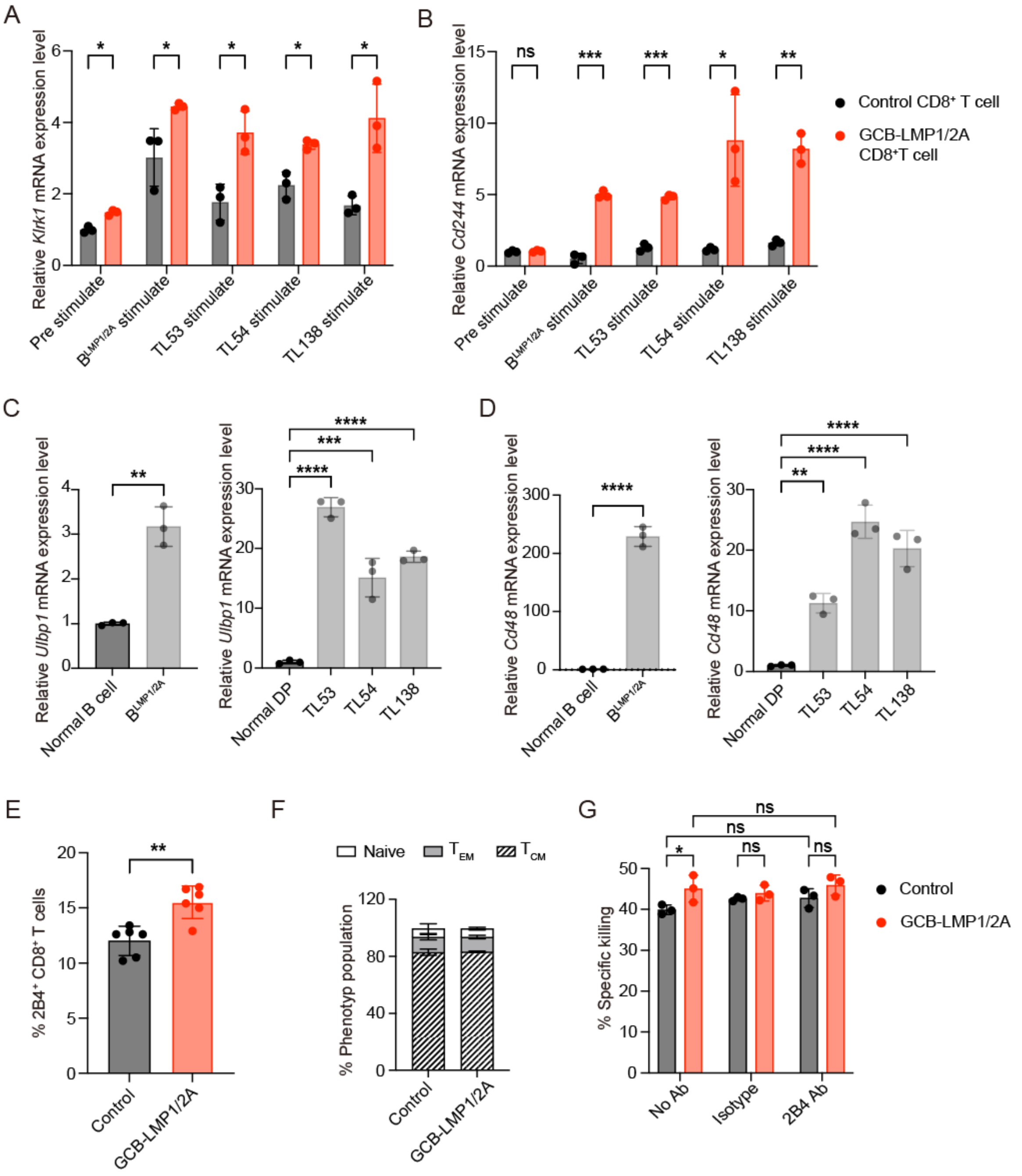

Supplementary Figure 5. Expression of cytotoxicity related genes in CD8$^+$ T cells and tumor cells and their impact on cytotoxic function

Relative mRNA expression levels of *Klrk1* **(A)** and *Cd244* **(B)** in CD8$^+$ T cells before stimulation and after 3 days of stimulation with B$^{LMP1/2A}$ cells or tumor cells. Data from three technical replicates is shown. Statistical analysis was performed using multiple t-tests, with Holm-Šidák correction for multiple comparisons; *$p < 0.05$; **$p < 0.01$; ***$p < 0.001$; ns, not significant. **(C, D)** Relative mRNA expression levels of *Ulbp1* **(C)** and *Cd48* **(D)** in normal B cells, B$^{LMP1/2A}$ cells, normal DP cells, and tumor cells. **(E)** 2B4 expression levels on CD8$^+$ T cells from GCB-LMP1/2A and control mice after co-culture with B$^{LMP1/2A}$ cells on day 3. **(F)** Population of various phenotypes within the 2B4$^+$ CD8$^+$ T cells. **(G)** Killing assay of TL53 cells with no antibody (No Ab), IgG2a isotype antibody (Isotype) and 2B4 antibody (2B4 Ab). Data represent the mean ± SD from three replicates. Statistical analysis was performed using two-way ANOVA with Bonferroni's multiple comparisons test and unpaired two-tailed Student's t-test; *$p < 0.05$; **$p < 0.01$; ***$p < 0.001$; ****$p < 0.0001$; ns, not significant.
